# *e*MatchSite: Sequence Order-Independent Structure Alignments of Ligand Binding Pockets in Protein Models

**DOI:** 10.1371/journal.pcbi.1003829

**Published:** 2014-09-18

**Authors:** Michal Brylinski

**Affiliations:** 1 Department of Biological Sciences, Louisiana State University, Baton Rouge, Louisiana, United States of America; 2 Center for Computation & Technology, Louisiana State University, Baton Rouge, Louisiana, United States of America; UCSD, United States of America

## Abstract

Detecting similarities between ligand binding sites in the absence of global homology between target proteins has been recognized as one of the critical components of modern drug discovery. Local binding site alignments can be constructed using sequence order-independent techniques, however, to achieve a high accuracy, many current algorithms for binding site comparison require high-quality experimental protein structures, preferably in the bound conformational state. This, in turn, complicates proteome scale applications, where only various quality structure models are available for the majority of gene products. To improve the state-of-the-art, we developed *e*MatchSite, a new method for constructing sequence order-independent alignments of ligand binding sites in protein models. Large-scale benchmarking calculations using adenine-binding pockets in crystal structures demonstrate that *e*MatchSite generates accurate alignments for almost three times more protein pairs than SOIPPA. More importantly, *e*MatchSite offers a high tolerance to structural distortions in ligand binding regions in protein models. For example, the percentage of correctly aligned pairs of adenine-binding sites in weakly homologous protein models is only 4–9% lower than those aligned using crystal structures. This represents a significant improvement over other algorithms, e.g. the performance of *e*MatchSite in recognizing similar binding sites is 6% and 13% higher than that of SiteEngine using high- and moderate-quality protein models, respectively. Constructing biologically correct alignments using predicted ligand binding sites in protein models opens up the possibility to investigate drug-protein interaction networks for complete proteomes with prospective systems-level applications in polypharmacology and rational drug repositioning. *e*MatchSite is freely available to the academic community as a web-server and a stand-alone software distribution at http://www.brylinski.org/ematchsite.

This is a *PLOS Computational Biology* Software Article

## Introduction

The ability of proteins to perform their molecular functions often associates with the reversible binding of a variety of small molecules, e.g. metabolites, neurotransmitters, hormones, and peptides. Ligand binding occurs on specific interaction sites, where depressions and pockets are formed at a protein molecular surface to facilitate binding through various non-covalent intermolecular forces including hydrogen bonds, electrostatic, and van der Waals interactions. These direct protein-ligand contacts along with the solvation and desolvation effects play a key role in the association process determining the strength of binding, or binding affinity [1]. Importantly, the specificity of binding sites towards small molecules arises from their chemical composition as well as geometric features. Many disease conditions can be directly linked to the cellular activities of certain molecular targets, modulating of which can restore homeostasis. Therefore, altering molecular functions of proteins using high-affinity compounds is a key strategy in pharmacotherapy. In particular, structure-based drug discovery involves the development and further optimization of synthetic and semi-synthetic compounds to target specific proteins of pharmacological relevance [2], [3]. Since modern drug discovery is routinely supported by computational approaches, such as virtual screening [4], [5] and quantitative structure-activity relationship methods [6], [7], the accurate modeling of protein-ligand interactions is of a paramount importance for the development of new and effective biopharmaceuticals.

Selectivity of binding remains a salient issue in pharmacology. Selective compounds have a tendency to bind to a limited number of different molecular targets in the cell, whereas those more promiscuous may affect the activity of a larger group of proteins often leading to adverse effects. The classical picture of very selective drug binding has been challenged by recent experimental and computational studies, which strongly suggest that the space of protein-drug interactions is dense and highly connected [8]. Several independent studies attempted to estimate the promiscuity of protein-drug interactions; for instance, a large-scale across-target activity analysis carried out for 189,807 active compounds from PubChem revealed that the majority (62%) of them exhibit activity against multiple, often unrelated targets [9]. Furthermore, a similar study conducted using a set of 3,138 compounds tested on up to 79 targets reported that 47% and 24% of the compounds can be classified as “promiscuous” and “highly promiscuous”, respectively, with multiple targets hit at the IC_50_ of <10 µM [10]. Finally, a thorough survey carried out for a network of 5,215 drug-target interactions connecting 829 drugs with 557 targets estimated that the average number of target proteins per drug is as high as 6.3 [11]. These numbers clearly indicate a high complexity of the protein-drug interaction space, however, most of the available data cover only a small subset of the “druggable” human proteome, which likely consists of >3,000 drug targets [12]. Moreover, the interaction space is covered non-uniformly with a couple of hundreds of the most actively pursued targets covering 90% of the testing compounds [13].

Clearly, new approaches that can address these issues and effectively support modern drug discovery are needed. Over the past decade, we observed a growing interest in computational methods that could give insights into the nature of protein-drug interactions. Classical algorithms for the detection of relationships between proteins widely used in bioinformatics and structural bioinformatics cannot be applied to explore drug cross-reactivity because many compounds bind to multiple proteins that are completely unrelated to each other at the global sequence and structure levels. For example, celecoxib, an inhibitor of cyclooxygenase-2, exhibit nanomolar affinity to an unrelated enzyme, carbonic anhydrase [14]. Telmisartan, an angiotensin II receptor antagonist used in the management of hypertension also acts as a partial agonist of the peroxisome proliferator-activated receptor-γ that regulates fatty acid storage and glucose metabolism [15]. Therefore, investigating drug cross-reactivity requires a different set of tools. Many of these explore ligand chemistry [16], similarity of gene expression profiles [17] or literature-mined side effects [18]. A direct comparison of binding sites has the capability to describe ligand binding at the molecular level providing useful insights into the drug mode of action. On that account, it is considered one of the most promising computational tools in computer-aided drug design and the prediction of biological function [19].

Most of the algorithms for binding site matching fall into one of two categories: alignment-free and alignment-based methods. Geometric hashing is a typical example of the alignment-free approach; it measures the overall similarity of two binding sites, however, without providing structural information on the putative ligand binding mode and its molecular interactions with the target protein. For instance, PocketMatch represents binding sites as the sorted lists of inter-residue distances that capture their shapes and chemical properties [20]. The comparison of binding sites is performed in a frame invariant manner by aligning the distance lists rather than residue coordinates. A pocket similarity is then computed based on the overlap between two ordered sequences of distances. Another example is SitesBase, a binding sites database that allows for a rapid retrieval of similar pockets, regardless of the global protein sequence and fold similarities [21]. Here, the underlying algorithm uses geometric matching at the level of atomic triplets to detect common features through the identification of cliques and maximum common sub-graphs; the similarities between local environments indicate both structural and functional relationships [22]. Templates used in the geometric hashing-based comparison of ligand binding pockets can be automatically derived from protein structures as demonstrated in the TESS program [23]. This algorithm employs a grid representation of functionally relevant sites, constructed based on reference frames defined individually for each of the 20 standard amino acid side chains. Surrounding atoms within a user-defined distance are first assigned to grid points; subsequently, the grid positions and the corresponding atomic labels are converted into a hash table for a rapid database searching. Templates automatically derived by TESS for the catalytic triad of ribonucleases and lysozymes have been used to identify several functionally interesting hits in the Protein Data Bank (PDB) [24].

In contrast to alignment-free techniques, methods based on binding site alignments elucidate why two sites are similar, identify the sets of atoms/residues that contribute to the similarity and describe putative ligand binding modes. However, a direct comparison of binding sites is more complicated and requires reliable sequence order-independent alignment techniques. Several such methods have been reported recently; for instance, SOIPPA performs sequence order-independent profile-profile alignments of binding pockets using a coarse-grained representation of protein structures [25]. This algorithm integrates geometric, evolutionary and physical information into a unified framework and assesses the alignment significance using the extreme value distribution model [26]. SuMo (Surfing the Molecules) was one of the first approaches to use a residue-independent stereochemical group description combined with a fast graph-based comparison heuristic to compare protein structures and substructures [27]. Its successor, MED-SuMo, was significantly improved to include functional annotation capabilities, new chemical features and a cavity-detection algorithm [28]. The effectiveness of MED-SuMo in detecting binding sites with similar structure-activity profiles was demonstrated using a large dataset of purine-binding proteins [29]. Another method, SiteEngine, employs low-resolution molecular surfaces constructed by converting triangles of physicochemical properties into a discrete set of chemically important surface points [30]. Assuming no sequence and fold similarity, SiteEngine offers hierarchical scoring schemes for global, local and global-local surface matching between proteins. A similar approach, ProBiS, recognizes structurally similar sites by analyzing patterns of physicochemical properties on the protein surface [31]. Using a fast maximum clique algorithm, this method also performs the comparison of complete protein surfaces. A clique-detection algorithm is also implemented in Cavbase [32] to compare cavities identified by Ligsite [33] using the degree of overlap between their exposed physicochemical properties. Cavbase employs 3D descriptors in the form of pseudocenters representing points important for molecular recognition, e.g. hydrogen bonds, hydrophobic and hydrophilic contacts. The application of Cavbase to the human kinome created a “binding pocket space”, which was shown to be highly effective in rationalizing the cross-reactivity between unrelated kinases [34]. In contrast to a sequence-based classification, which is often unable to detect cross-relations between individual kinases, approaches such as Cavbase provide useful insights to support the development of more selective drugs.

Ligand binding sites can be represented by “clouds” of atoms having certain properties, e.g. types, partial charges, etc., as implemented in the sup-CK algorithm [35]. Sup-CK assesses the similarity between two pockets using a convolution kernel upon the optimal alignment of their atomic “clouds”. A recently developed method, TIPSA, employs the iterative closest point algorithm to superpose and compare binding pockets using the atom-level representation of protein surfaces [36]. The maximum number of superposable atoms between two binding sites is identified based on the initial local alignments derived from 3D Delaunay triangulations. To increase the prediction accuracy, TIPSA incorporates additional global geometric information, the radius of gyration of binding site atoms, and an effective nearest neighbor classification scheme. Another example of a method that employs sequence order-independent alignments of binding surfaces is Solar (Signature Of Local Active Regions) [37]. This approach introduces a concept of signature binding sites and signature basis sets designed to capture information about the conserved and variable atomic positions at multi-resolution levels. Interesting features of Solar include hierarchically organized degrees of partial structural similarity, and an effective procedure for the identification of residues and atoms that are important for binding affinity and specificity, as demonstrated for metalloendopeptidase enzymes. Despite the encouraging progress in the development of sequence order-independent algorithms for ligand binding site alignment, many of these approaches require high-quality binding sites extracted from either experimental protein structures complexed with ligands or close homology models constructed using holo-templates in order to achieve a high accuracy.

To mitigate this issue, we developed *e*MatchSite, a new algorithm that performs sequence order-independent local binding site alignments using computer-generated protein models. In addition to its high tolerance to distortions in the target structures, *e*MatchSite also aligns predicted ligand binding sites that may contain inaccuracies in the definition of binding residues. A key feature responsible for its high performance is the extensive use of evolutionary information that can be extracted from only weakly homologous templates complexed with ligands. Essentially, the current work extends ideas already explored in binding pocket prediction by algorithms such as FINDSITE [38] and its successor, *e*FindSite [39], to address the problem of aligning and quantifying the similarities between ligand binding sites in proteins. The performance of *e*MatchSite is evaluated using several datasets and compared to other algorithms for binding site matching in large-scale benchmarking calculations. The results demonstrate that *e*MatchSite maintains its high prediction accuracy against protein models, which should prove useful in systems-level applications, such as polypharmacology and rational drug repositioning.

## Design and Implementation

*e*MatchSite is a sequence-order independent algorithm for ligand binding site alignment and comparison. It employs a set of residue-level scores extracted from weakly homologous template proteins complexed with small molecules that cover various properties of binding ligands and residues. Evolutionary information is included as sequence profiles and entropy, as well as secondary structure profiles. Hydrophobicity parameters for amino acids, the spatial distribution of residues and ligand binding probabilities capture physicochemical and structural characteristics of protein residues and their interactions with small molecules. An important component is the chemical matching of template-bound ligands that effectively explores the conservation of binding site chemistry and ligand binding geometry across sets of functionally related proteins. Individual scores are combined using non-linear machine learning models and the alignments of binding sites are constructed by the Kuhn-Munkres algorithm [40], [41] (also known as the Hungarian method) for solving assignment problems.

Validation of the fold-independent matching of ligand binding sites requires specific datasets of proteins that bind chemically similar ligands despite having different sequences and structures. In this study, we use four datasets, the SOIPPA dataset of adenine-binding proteins [25], the Kahraman and Homogeneous datasets comprising a variety of small molecules [35], [42], and the Steroid dataset of pharmacologically relevant steroid-binding proteins. In addition to the crystal structures of target proteins, we constructed high- and moderate-quality models to assess the performance of binding site matching using computer-generated structures. Moreover, we focus on predicted binding sites that may contain some inaccuracies in binding residue definition rather than experimental pockets.

The performance of *e*MatchSite is compared to several other predictors, SOIPPA [25], PocketMatch [20], SiteEngine [30] and sup-CK [35]. These approaches represent a variety of computational techniques developed to compare ligand binding sites in proteins, including geometric hashing, surface-based methods and sequence order-independent profile-profile alignments. Local predictors are also compared to two naïve approaches that employ global sequence and structure alignments of target proteins. Using global similarity helps detect any possible bias that may be present in a particular dataset, i.e. pairs of proteins that bind similar ligands may also be related at the global sequence and/or structure level making them relatively easy targets. In the subsequent sections, we provide a detailed description of the datasets, *e*MatchSite implementation, evaluation metrics, and validation protocols used in this study.

### Datasets

The primary dataset used in this study to train and cross-validate machine learning models implemented in *e*MatchSite comprises adenine-binding proteins as well as control proteins that do not bind ligands containing the adenine moiety. This dataset was compiled previously to benchmark the performance of another binding site alignment algorithm, SOIPPA [25]. According to the SCOP classification [43], target proteins represent 167 superfamilies and 146 folds. Ligands included in this dataset are adenosine-5′-diphosphate (ADP), adenosine-5′-triphosphate (ATP), flavin-adenine dinucleotide (FAD), nicotinamide-adenine-dinucleotide (NAD), S-adenosyl-L-homocysteine (SAH), and S-adenosylmethionine (SAM). Control ligands in the SOIPPA dataset form 48 chemically representative clusters at a Tanimoto coefficient [44] threshold of 0.7.

In addition, we assess the performance of binding site matching using two other datasets. The Kahraman dataset was previously developed to analyze the shapes of protein binding pockets with respect to the shapes of their ligands [42]. This dataset comprises proteins bound to adenosine monophosphate (AMP), 3-β-hydroxy-5-androsten-17-one (AND) adenosine-5′-triphosphate (ATP), estradiol (EST), flavin-adenine dinucleotide (FAD), flavin mononucleotide (FMN), α-D-glucose (GLC), protoporphyrin IX containing Fe (HEM), and nicotinamide-adenine-dinucleotide (NAD). The Homogeneous dataset was compiled to benchmark the performance of sup-CK, a method to quantify the similarity between binding pockets [35]. It consists of proteins complexed with the following ligands: pentaethylene glycol (1PE), B-octylglucoside (BOG), glutathione (GSH), lauryl dimethylamine-N-oxide (LDA), palmitic acid (PLM), 4′-deoxy-4′-aminopyridoxal-5′-phosphate (PMP), S-adenosylmethionine (SAM), sucrose (SUC), and uridine-5′-monophosphate (U5P). Although some ligands, e.g. 1PE and BOG, may bind non-specifically to proteins and are used to facilitate the crystallization process, we keep them in the dataset to make the results comparable to those reported in the original publication [35]. When assessing the performance using the Kahraman and Homogeneous datasets, positives are defined as pairs of proteins that bind exactly the same ligand, whereas those proteins that bind different ligands are considered negatives.

The last dataset contains 8 pharmacologically relevant steroid-binding proteins complexed with 17β-estradiol (EST), estradiol-17β-hemisuccinate (HE7), and equilenin (EQU). As the control dataset, we use 1,854 proteins that bind small molecules whose size is comparable to that of steroids (15–25 heavy atoms), however, these ligands have different chemical structures with a Tanimoto coefficient [44] vs. EST of ≤0.1. Control ligands in the Steroid dataset form 334 chemically representative clusters at a Tanimoto coefficient threshold of 0.7. According to the SCOP classification [43], target proteins represent 185 superfamilies and 150 folds.

### Target structures

In addition to the crystal structures of target proteins, we constructed weakly homologous protein models for the SOIPPA, Kahraman, Homogeneous and Steroid datasets. The models were assembled using template-based modeling by *e*Thread [45], [46], excluding those templates whose sequence similarity to the target is >40%. First, we built up to 20 models for each target, 10 using *e*Thread/Modeller and 10 using *e*Thread/TASSER-Lite. Then, one model with a TM-score to native of >0.7 was randomly selected and included in the high-quality dataset. Similarly, a randomly selected model with a TM-score of 0.4–0.7 was included in the moderate-quality dataset. Other than crystal structures and weakly homologous models, the SOIPPA dataset also comprises artificially distorted structures, whose Cα-RMSD is within a narrow range (RMSD stands for a root-mean-square deviation). These structures were constructed by distorting the native conformation using an in-house software that employs conformational Monte Carlo sampling to reach the desired RMSD from native while preserving the secondary structure content [47]. Specifically, for each target in the SOIPPA dataset, we built three non-native structures with a Cα-RMSD of 2 Å, 4 Å and 6 Å.

### Ligand binding site prediction

Ligand binding sites were identified in target proteins using *e*FindSite, a recently developed template-based approach [39], [48]. Similar to structure modeling, binding pocket prediction was performed using only weakly homologous templates with a sequence identity to the target of ≤40%. In pocket matching calculations, we used only those proteins, for which the center of each of the best of top five binding sites is predicted within a distance of 8 Å from the experimental pocket center, with the corresponding Matthew's correlation coefficient calculated over binding residues of ≥0.4. The accuracy of ligand binding site prediction certainly depends on the quality of target structures [39], therefore, as shown in Tables 1 and S1, the structural subsets of the SOIPPA, Kahraman, Homogeneous and Steroid datasets (crystal structures, high- and moderate-quality models as well as distorted conformations) comprise different numbers of proteins.

**Table 1:** Global and local structure quality of adenine-binding proteins from the SOIPPA dataset.

Dataset	Number of targets	Global structure		Ligand binding pocket		
		*Cα-RMSD [Å]*	*TM-score*	*RMSD* a *[Å]*	*Distance* b *[Å]*	*MCC* c
Crystal structures	211	-	-	-	1.7±1.4	0.70±0.10
High-quality models	202	4.4±2.4	0.83±0.07	2.0±1.4	1.8±1.4	0.67±0.10
Moderate-quality models	174	13.2±4.6	0.54±0.10	5.7±3.5	1.9±1.3	0.62±0.10

a Heavy-atom RMSD calculated over binding residues.

b Distance between predicted pocket center and the geometric center of bound ligand.

c Matthew's correlation coefficient for predicted binding residues.

High- and moderate-quality models are constructed by *e*Thread. Ligand binding sites and residues are detected by *e*FindSite.

### Implementation of *e*MatchSite

A unique feature of *e*MatchSite is its capability to estimate pairwise Cα-Cα distances between binding residues upon the alignment of two pockets using machine learning and a set of seven residue-level scores. These features cover various physicochemical and geometric characteristics and, importantly, can be extracted from only weakly homologous template structures identified by *e*FindSite. Residue-level scores implemented in *e*MatchSite employ sequence and secondary structure profiles, hydrophobicity parameters for amino acids, ligand binding probabilities, the spatial distribution of neighboring residues, sequence entropy, and the chemical matching of template-bound ligands.

**Sequence profile score**. For each target protein, a sequence profile is constructed using PSI-BLAST [49] and a non-redundant sequence database (nr) from NCBI [50]. The nr database was filtered to remove low-complexity regions, transmembrane and coiled-coil segments [51]. Given a pair of residues *i* and *j*, the sequence profile score, 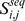, is the dot product of their profile vectors:
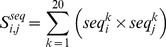
(1)where 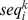 is the value for the amino acid *k* in the *i*-th position of the sequence profile of the first protein, and 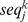 is the value for the amino acid *k* in the *j*-th position of the sequence profile of the second protein.

**Secondary structure score**. PSIPRED [52] is used to assign three probability values to each residue corresponding to an α-helix, a β-structure and a loop conformation. The secondary structure score for a pair of residues *i* and *j*, 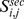, is the Euclidean distance between their secondary structure probability vectors.
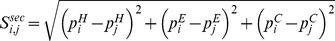
(2)where 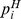, 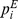 and 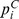 are, respectively, the probability for α-helix (*H*elix), β-structure (*E*xtended) and loop (*C*oil) assigned by PSIPRED to the *i*-th residue in the first protein. 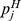, 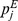 and 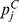 are the equivalent values for the *j*-th residue in the second protein.

**Hydrophobicity score**. Each residue type is assigned a vector of 20 hydrophobicity parameters according to the following experimental and theoretical hydrophobicity scales for amino acids: Abraham and Leo [53], Black and Mould [54], Brylinski *et al.* [55], Bull and Breese [56], Cowan and Whittaker [57], Eisenberg *et al.* [58], Fauchere and Pliska [59], Guy [60], Hopp and Woods [61], Janin [62], Kyte and Doolittle [63], Manavalan *et al.* [64], Miyazawa and Jernigan [65], Parket *et al.* [66], Rao and Argos [67], Roseman [68], Tanford [69], Welling *et al.* [70], Wilson *et al.* [71], and Wolfenden *et al.* [72]. The hydrophobicity score, 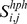, corresponds to the Pearson correlation coefficient calculated between two hydrophobicity vectors for residues *i* and *j*:
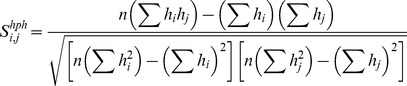
(3)where *n* is the number of hydrophobicity scales (20), *h_i_* and *h_j_* are hydrophobicity parameters for residues *i* (first protein) and *j* (second protein), respectively. The summations of hydrophobicity parameters (*h_i_* and *h_j_*), squared (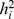 and 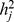) and paired (*h_i_h_j_*) values are taken over 20 hydrophobicity scales.

**Binding probability score**. *e*FindSite assigns a ligand binding probability to each predicted binding residue in the protein target [39]. The binding probability score, 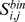, is a squared difference between the binding probabilities assigned to a pair of residues *i* and *j*:
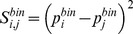
(4)where 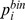 and 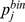 is a ligand binding probability assigned by *e*FindSite to *i*-th residue in the first protein and *j*-th residue in the second protein, respectively.

**Neighbor distribution score**. For each binding residue, we first calculate the distribution of Cα distances to all other residues in the same pocket:
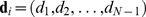
(5)where **d** *_i_* is a vector of distances between *i*-th residue and the remaining binding residues in the first protein, enumerated from *d*_1_ to *d_N_*_-1_; **d** *_j_* is the equivalent vector for the *j*-th residue in the second protein.

Then, given a pair of residues *i* and *j* belonging to different pockets, we compare their neighbor distance distributions, **d** *_i_* and **d** *_j_*, using the non-parametric Fisher-Pitman permutation test for independent samples [73]. The T-value returned by this test is used as the neighbor distribution score, 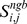.

**Sequence entropy score**. From sequence profiles generated by PSI-BLAST, the amino acid variability at a given residue position is quantified using the Shannon entropy, which provides a simple measure of uncertainty in a data set [74]. The sequence entropy score, 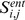, is a squared difference between individual entropies calculated for a pair of residues *i* and *j*:
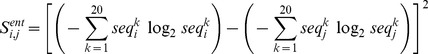
(6)where 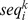 and 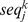 have the same meaning as in Eq. 1.

#### Template ligand score

*e*FindSite predicts binding sites using evolutionarily related holo-templates. Template structures are superposed onto a target protein and template-bound ligands are transferred to the target upon the global structure alignment. *e*MatchSite uses these ligands to position two target proteins relative to each other. Specifically, atomic equivalences are established between two template ligands (one from each target protein) using kcombu, a heuristic build-up algorithm for determining one-to-one atom correspondences between chemical compounds [75]. Next, the two target proteins are oriented in space according to the superposition of template ligands and pairwise Cα-Cα distances between binding residues in the targets are calculated. We repeat this procedure *m*×*n* times, where *m* and *n* are the number of template ligands collected by *e*FindSite for the first and the second target protein, respectively. Given a pair of binding residues *i* and *j* from both targets, the template ligand score, 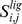, is a weighted average distance between their Cα atoms calculated for all template ligand combinations:
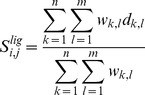
(7)where a weight *w_k_*_,*l*_ corresponds to the squared Tanimoto coefficient [44] between template ligands *k* and *l* reported by kcombu. Thus, the contribution from highly similar ligand pairs is larger than from those chemically less similar. *d_k_*_,*l*_ is a distance between the Cα atoms of residues *i* in the first protein and *j* in the second protein when their structures are oriented according to the alignment of template ligands *k* and *l*.

Note that the set of seven residue-level scores, 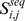, 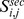, 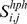, 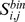, 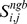, 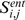 and 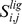, are calculated for putative binding sites identified by *e*FindSite without using any information on the actual target-bound ligands. Therefore, this procedure can be applied to experimental structures in their apo conformations as well as to computer-generated protein models. Next, we constructed a machine learning model to estimate distances between the Cα atoms of residues belonging to the two target pockets upon their optimal local alignment. Reference distances are calculated upon the superposition of protein structures using the coordinates of bound ligands. SVR is used to predict these distances using the set of seven residue-level scores; here, we use the SVR implementation from libSVM 3.14 [76]. Machine learning model is cross-validated against the SOIPPA dataset. We use a non-exhaustive 6-fold cross-validation, where a subset of dataset proteins binding to a particular ligand are excluded, the model is trained on the remaining cases and Cα-Cα distances are predicted for the excluded group. This procedure is applied to all 6 ligands in the SOIPPA dataset. In addition to the SVR model, we also evaluated an equivalent procedure employing SVC using the same set of seven residue-level scores. Here, rather than estimating Cα-Cα distances, the model predicts whether a pair of binding residues align to each other upon the optimal local superposition of two binding sites. We found that the algorithm based on the SVR model performs slightly better than that using SVC, therefore the latter was not pursued further.

Using the machine learning-based procedure described above, we calculate an all-against-all matrix containing the estimated Cα-Cα distances between residues belonging to two target pockets. The optimal alignment is found by applying the Kuhn-Munkres algorithm [40], [41] to identify a unique set of residue pairs that give the shortest overall distance between their Cα atoms. This technique, also known as the Hungarian method, solves combinatorial assignment problems in polynomial time. The sum of Cα-Cα distances for the solution is guaranteed to be the smallest amongst all possible alignment combinations. Moreover, this algorithm produces fully sequence order-independent alignments, whose length is equal to the number of binding residues in the smaller pocket.

### Pocket similarity score

Finally, optimal alignments of pairs of ligand binding pockets are assigned a similarity score corresponding to the probability that these sites bind similar ligands. The similarity score is calculated using machine learning and an input vector of the following features: a Cα-RMSD calculated over equivalent binding residues, average residue-level scores, a chemical correlation, the physicochemical properties of putative binding ligands, and geometric hashing.

#### Pocket RMSD

The geometric fit between two pockets, *F^RMS^*, corresponds to the minimum Cα-RMSD calculated for residue equivalences from the optimal alignment.

#### Average residue-level scores

In addition to the actual RMSD between two pockets, we include the predicted SVR and SVC scores averaged over aligned residue pairs, *F^SVR^* and *F^SVC^*:
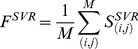
(8)

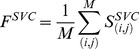
(9)where the sum is taken over *M* aligned residue pairs (*i*,*j*) between the two proteins. 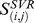 is a score reported by SVR that corresponds to the expected distance between Cα atoms of equivalent binding residues (*i*,*j*) and 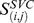 is a score reported by SVC that gives the probability that residues (*i*,*j*) align to each other.

#### Chemical correlation

*e*FindSite employs molecular fingerprints constructed for ligands extracted from evolutionarily related templates to conduct ligand-based virtual screening against predicted binding pockets [48]. *e*MatchSite uses this capability to perform virtual screening against the two predicted pockets and calculates the Kendall τ rank correlation coefficient, *F^TAU^*:
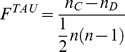
(10)where *n_C_* and *n_D_* are the numbers of concordant and discordant pairs, respectively; the denominator is the total number of pair combinations. Any pair of library compounds is concordant if their ranks in the ordered lists for the two pockets agree, i.e. one compound is consistently ranked higher than the other. Pairs of compounds whose relative ranks are swapped in the two ordered lists are considered discordant. To perform virtual screening, we compiled a small library of 23,659 molecules selected from the ZINC collection of organic compounds by removing the redundancy at a Tanimoto coefficient [44] threshold of 0.5 using the SUBSET program [77]. The chemical correlation was formulated previously to construct a cross-reactivity virtual profile for the human kinome [78].

#### Physicochemical properties

Each ligand binding site identified by *e*FindSite is also assigned a set of consensus physicochemical properties of putative binding ligands, including the molecular weight (MW), the octanol/water partitioning coefficient (logp), the polar surface area (PSA), and the number of hydrogen bond donors and acceptors (HBD and HBA, respectively) [39]. As a physicochemical feature, *F^PCF^*, we average the differences between two binding pockets with respect to these properties:
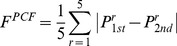
(11)where the sum is taken over the five abovementioned physicochemical properties, and *P*_1*st*_ and *P*_2*nd*_ are the binding pockets in the first and second protein, respectively.

#### Geometric hashing

The last feature is an alignment-free matching score calculated using geometric hashing. Here, we implemented in *e*MatchSite a scoring scheme from PocketMatch, which represents each binding site as a sorted list of 90 distances between Cα, Cβ atoms, and the side chain geometric centers for amino acid residues arranged into 5 groups: group-0: A, V, I, L, M, G, P; group-1: K, R, H; group-2: D, E, Q, N; group-3: Y, F, W; and group-4: C, S, T [20]. The pairs of distance-sets are aligned using a greedy strategy and the similarity score is calculated as the average fraction of matching elements across the sorted lists of distances. This feature in *e*MatchSite is denoted by *F^PMS^*, where PMS stands for the original PocketMatch score [20].

The pocket similarity score is computed by combining the six features described above using machine learning. The training and validation of the machine learning model used to assess similarities between pairs of pockets is carried out using adenine-binding proteins from the SOIPPA dataset. We follow a similar 6-fold cross-validation protocol as described above for assessing the inter-residue distance prediction. Machine learning for the estimation of pocket similarity is implemented using the Support Vector Machines algorithm for classification problems provided by libSVM 3.14 [76].

### Evaluation metrics

The quality of local binding site alignments is assessed against reference alignments using Matthew's correlation coefficient (MCC):
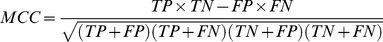
(12)where *TP*, *FN* and *FP* are the number of correctly aligned residue positions, under- and overpredicted, respectively. *TN* is the number of residue pairs correctly predicted not to align to each other. Reference alignments are constructed by superposing a pair of protein structures using the coordinates of bound ligands. We note that similar pockets in the Kahraman and Homogeneous datasets are defined as those that bind the same ligand, whereas in the SOIPPA and Steroid datasets, similar pockets bind ligands containing the adenine and estradiol moieties, respectively. Here, the superposition is performed using the maximum common substructures between two ligands identified by the Small Molecule Subgraph Detector (SMSD) [79]. Upon the superposition, the reference alignment is calculated by applying the Hungarian algorithm [40], [41] to a matrix of all-against-all distances between binding residue Cα atoms (a similar procedure is described in [36]). Subsequently, an optimal structure alignment of two binding sites is constructed, where the alignment length is equal to the number of residues in the smaller pocket. This algorithm guarantees that the sum of Cα-Cα distances calculated over aligned residue positions is the smallest amongst all possible alignments with the same length.

The alignment quality is further assessed by a ligand heavy-atom RMSD with an underlying assumption that the correct alignment of binding residues would prompt two ligands to adopt a similar orientation. Specifically, we superpose two proteins using residue Cα atoms based on a given local binding site alignment, which is followed by calculating an RMSD for bound ligands. The SOIPPA, Kahraman and Homogeneous datasets contain flexible compounds with multiple rotatable bonds that may have different internal geometries when bound to different proteins. Therefore, we use a method for correcting the RMSD by subtracting a heavy-atom RMSD calculated upon the superposition of two ligands alone; this corrected metric is denoted by ΔRMSD.

In addition to the quality of local binding site alignments, we assess the capabilities of different algorithms to detect those pockets binding similar ligands. The SOIPPA dataset comprises two groups of structures, adenine-binding proteins and control proteins that do not bind ligands containing the adenine moiety. Here, positives are defined as pairs of adenine-binding proteins, whereas pairs of an adenine-binding protein and a control protein are considered negatives. An analogous definition of positives and negatives is used for the steroid-binding and control proteins in the Steroid dataset. For the Kahraman and Homogeneous datasets, positives and negatives are pairs of proteins that bind the same and different ligands, respectively. The ability to detect similar binding sites in different proteins is assessed by a receiver operating characteristics (ROC) and the corresponding area under the ROC curve (AUC). In this analysis, a true positive rate (TPR, also called sensitivity) and a false positive rate (FPR, also called fall-out) are defined as:
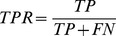
(13)

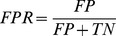
(14)where *TP*, *TN*, *FP* and *FN* are the numbers of true positives, true negatives, false positives and false negatives, respectively.

### Other predictors

The accuracy of *e*MatchSite is compared to that of several other methods. The first two represent global sequence and structure alignment approaches. Sequence alignments between two proteins are calculated by Needleman-Wunsch dynamic programming [80] with a sequence identity used as the alignment score. Global structure alignments are performed by Fr-TM-align [81], where the alignment significance is evaluated by a TM-score [82]. In addition to these global similarity measures, we analyze the performance of *e*MatchSite with respect to various local binding site matching algorithms. PocketMatch represents an alignment-free, geometric hashing approach that implements a PMScore to measure the similarity between ligand binding sites [20]; the stand-alone version of PocketMatch 2.0 is used in this study. SiteEngine is a surface-based algorithm developed to recognize similar functional sites shared by proteins that have different sequences and folds [30]. It measures the similarity in terms of the overlap between the physicochemical and geometric properties of binding pockets. The stand-alone version of SiteEngine 1.0 was used in a binding site comparison mode. Sup-CK is a method that represents ligand binding pockets by clouds of atoms and assesses the pocket similarity using a convolution kernel upon the optimal superposition of their atomic clouds in space [35]. For each program, PocketMatch, SiteEngine and sup-CK, the calculations are conducted using the default set of parameters. Finally, SOIPPA is a protein functional site comparison algorithm that features sequence order-independent profile–profile alignments, which are calculated for a reduced representation of protein structures [25]. The comparison of *e*MatchSite to SOIPPA is performed only for the crystal structures of target proteins, using supplementary data reported in the original publication of SOIPPA.

## Results

### Characteristics of target structures

*e*MatchSite was devised specifically for applications involving protein models, therefore we first discuss the structural characteristics of dataset proteins used in this study. In addition to crystal structures, we perform local binding site alignment benchmarks using weakly homologous protein models and artificially distorted structures. The former are constructed using *e*Thread, a template-based approach to protein structure modeling [45], [46]. Table 1 shows the structure quality of protein models generated for the SOIPPA dataset. High- and moderate-quality models have an average TM-score to native of 0.83 and 0.54, respectively; this corresponds to the global Cα-RMSD (local binding pocket all-atom RMSD) of 4.4 Å (2.0 Å) for high- and 13.2 Å (5.7 Å) for moderate-quality models. Structures with a comparable quality were constructed for the Kahraman, Homogeneous and Steroid datasets; see Supporting Information, Table S1. Furthermore, to generate more uniform sets of non-native models, we distorted crystal structures to the desired RMSD with a small standard deviation. Table S1 shows that models deformed to 2 Å, 4 Å and 6 Å Cα-RMSD from native have an average TM-score of 0.91, 0.78 and 0.68, respectively; their binding sites are distorted to 1.3 Å, 2.4 Å and 3.2 Å all-atom RMSD.

In addition to the target structure, binding site matching also requires a pre-defined set of binding residues, which can be identified in experimental target structures complexed with small molecules. However, this information is unavailable for apo conformations and protein models. Therefore, an algorithm for binding site alignment should tolerate to some extent inaccuracies in the binding residue definition in order to incorporate predicted binding pockets. In that regard, we focus on binding sites predicted using recently developed *e*FindSite [39] rather than those obtained experimentally. Table 1 shows that the average distance between experimental and predicted pockets for the SOIPPA dataset is 1.7 Å, 1.8 Å and 1.9 Å for crystal structures, high- and moderate-quality models, respectively; the corresponding average Matthew's correlation coefficient (MCC) calculated for binding residues is 0.70, 0.67 and 0.62. As shown in Table S1, binding sites for the Kahraman, Homogeneous and Steroid datasets are predicted with a slightly lower accuracy; depending on the quality of target structures, the average distance is 2.0–2.2 Å, 2.9–3.2 Å and 2.3–2.5 Å, with the corresponding MCC of 0.59–0.65, 0.59–0.63 and 0.61–0.67, respectively.

**Figure 1:**
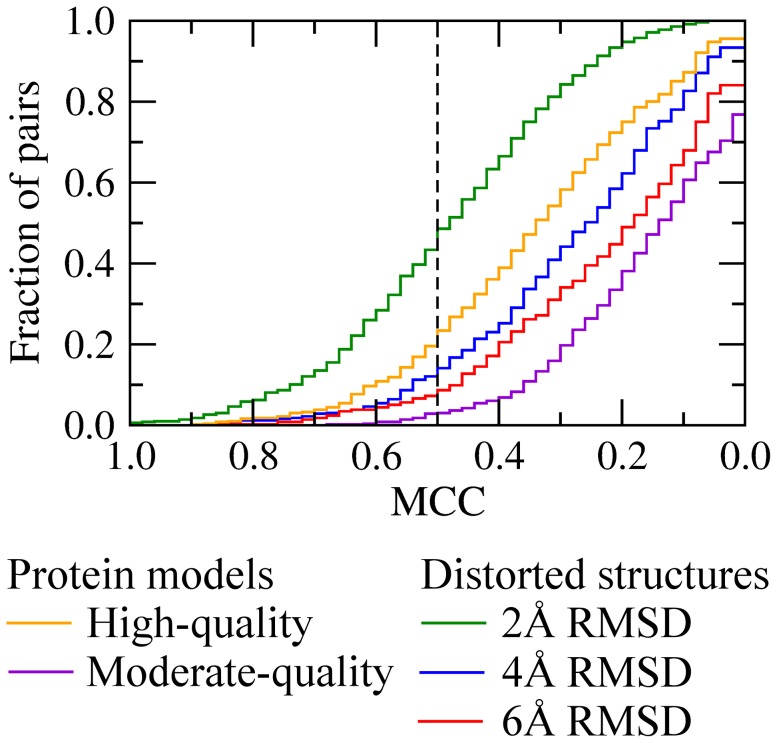
Effects of target structure distortions on the quality of local alignments of ATP-binding sites. MCC is Matthew's correlation coefficient calculated against the reference alignments constructed using target crystal structures.

We also investigate how structural imperfections in protein models affect the alignment of binding sites. For the SOIPPA dataset, we first derive reference alignments of binding pockets by superposing ligands bound to target crystal structures. Then, we repeat this procedure using binding sites predicted in protein models as well as distorted structures to assess the alignment accuracy by calculating MCC vs. the reference alignments. Figures 1 and S1 show that even minor structural imperfections combined with inaccuracies in binding residue prediction significantly alter the alignments. For instance, alignments constructed for 22.2%, 4.1%, 48.9%, 10.9% and 3.8% pairs of ATP-binding sites have MCC≥0.5 when high-, moderate-quality models, structures distorted to 2 Å, 4 Å and 6 Å are used (Figure 1). Qualitatively similar accuracy is obtained for other ligands in the SOIPPA dataset (Figure S1). This analysis indicates that non-native target structures pose significant challenges to algorithms for local ligand binding site alignment.

### Residue-level scores extracted from weakly homologous templates

**Figure 2:**
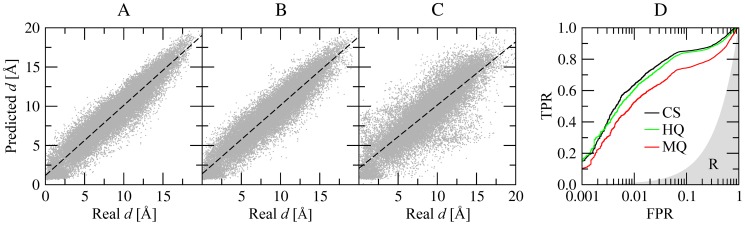
Prediction of aligned residue pairs using machine learning for SAH-binding proteins from the SOIPPA dataset. The correlation between the actual pairwise Cα-Cα distances upon the reference alignment of binding sites and those predicted by SVR is shown for (**A**) crystal structures, (**B**) high-, and (**C**) moderate-quality protein models, respectively. (**D**) The ROC plot for the prediction of equivalent residue pairs using SVC; CS – crystal structures, HQ – high-quality, MQ – moderate-quality models, R – random prediction.

*e*MatchSite constructs binding site alignments from all-against-all pairwise Cα-Cα distances estimated by machine learning using a set of residue-level scores. The accuracy of inter-residue distance prediction is critical for the alignment quality. For the SOIPPA dataset, Tables 2 and S2 show the Pearson correlation coefficients (PCC) between the actual distances upon the superposition of binding ligands and those predicted by Support Vector Machines (for regression problems, SVR). The corresponding correlation plots are presented in Figures 2 and S2. For example, PCC for proteins binding S-adenosyl-L-homocysteine (SAH) is 0.95, 0.94 and 0.86, when the Cα-Cα distances are predicted using crystal structures, high- and moderate-quality models, respectively (Table 2 and Figures 2A–C). In addition to SVR, we constructed another Support Vector Machines model (for classification problems, SVC), which predicts aligned pairs using the same set of residue-level scores. The accuracy of this classifier for SAH-binding proteins from the SOIPPA dataset is shown in Figure 2D; at a fixed false positive rate of 1%, the true positive rate is 63.6%, 60.6% and 52.6% for crystal structures, high- and moderate-quality models, respectively. The performance of the SVC model for other proteins is shown in Figure S3. These results demonstrate that residue-level scores extracted from evolutionarily weakly homologous templates can be used to accurately predict inter-residue distances for local binding site alignments. Furthermore, the SVR model performs slightly better than the SVC classifier in constructing the actual alignments, therefore the former is used as the default method in *e*MatchSite.

**Table 2:** Accuracy of inter-residue distance prediction for adenine-binding proteins from the SOIPPA dataset.

Structure dataset	Binding ligand											
	*ADP*		*ATP*		*FAD*		*NAD*		*SAH*		*SAM*	
	*PCC* a	*MSE* b	*PCC* a	*MSE* b	*PCC* a	*MSE* b	*PCC* a	*MSE* b	*PCC* a	*MSE* b	*PCC* a	*MSE* b
Crystal structures	0.79	6.4	0.73	8.1	0.96	2.4	0.89	4.6	0.95	1.6	0.76	7.2
High-quality models	0.75	7.3	0.72	8.6	0.92	4.6	0.86	6.1	0.94	1.9	0.88	3.6
Moderate-quality models	0.75	7.9	0.68	10.1	0.88	7.2	0.86	6.3	0.86	4.1	0.83	4.9

The Pearson correlation coefficient (PCC) and the mean squared error (MSE) are calculated for the actual pairwise Cα-Cα distances upon the superposition of binding ligands and those predicted by SVR from residue-level scores. The accuracy is reported separately for different binding ligands and target protein conformations including crystal structures, high- and moderate-quality protein models.

a Pearson correlation coefficient.

b Mean squared error in Å.

### Binding pocket alignment by *e*MatchSite

**Figure 3:**
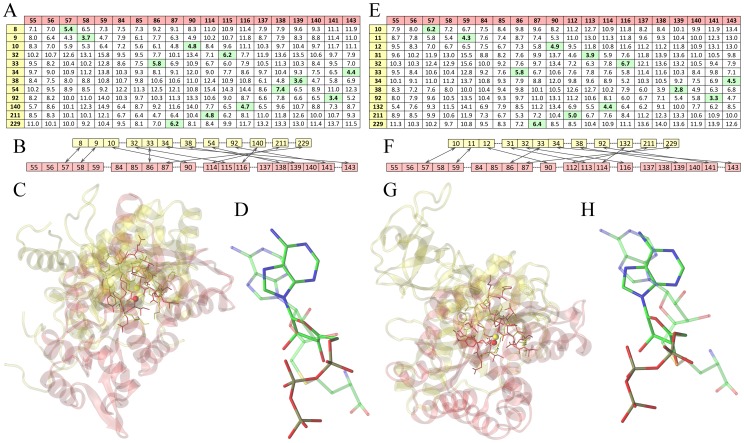
Construction of sequence order-independent binding site alignments by *e*MatchSite. Two target proteins are ATP-dependent DNA ligase (PDB-ID: 1a0iA, yellow) and histamine N-methyltransferase (PDB-ID: 2aotA, red). Left (**A**–**D**) and right (**E**–**H**) panels show the alignment of binding sites in the crystal structures and protein models, respectively. (**A**, **E**) Matrices of pairwise Cα-Cα distances between two binding sites predicted by SVR. Residue indexes are shown in the first column and row. Sets of residue pairs that have the smallest Cα-Cα distances identified by the Kuhn-Munkres algorithm are highlighted in green. (**B**, **F**) Sequence order-independent alignments of two binding sites constructed from residue pairs that have the smallest Cα-Cα distances; arrows indicate equivalent pairs. (**C**, **G**) Protein structures are superposed according to the local alignment of their binding sites; binding residues and predicted pocket centers are shown as solid sticks and balls, respectively. (**D**, **H**) Relative orientation of binding ligands upon the local alignment of target binding sites; ATP in 1a0iA and S-adenosyl-L-homocysteine in 2aotA are shown as solid and transparent sticks, respectively.

The algorithm for the sequence order-independent alignment of binding sites implemented in *e*MatchSite is illustrated in Figure 3 for two unrelated proteins, ATP-dependent DNA ligase (PDB-ID: 1a0iA) and histamine N-methyltransferase (PDB-ID: 2aotA). Both proteins bind ligands that contain the adenine moiety, ATP and S-adenosyl-L-homocysteine, respectively. However, they share little similarity at the global sequence and structure levels; their pairwise sequence identity is 23% and the TM-score between them is 0.28. Using crystal structures, the distance between the experimental pocket center and that predicted by *e*FindSite (MCC calculated over binding residues) for 1a0iA and 2aotA is 2.55 Å (0.81) and 1.86 Å (0.68), respectively. Figure 3A shows the matrix of all-against-all Cα-Cα distances estimated by machine learning using SVR, where the pairs of residues selected by the Kuhn-Munkres algorithm [40], [41] to minimize the overall distance are highlighted in green. These pairs are translated to the sequence order-independent alignment of binding residues presented in Figure 3B. Furthermore, Figure 3C shows the superposition of two target proteins according to the local alignment of their binding sites; the Cα-RMSD calculated over equivalent residue pairs is 2.13 Å. The alignment accuracy can be evaluated using the relative orientation of binding ligands upon the superposition of target proteins as shown in Figure 3D. In addition to experimental structures, Figures 3E–H show the performance of *e*MatchSite using weakly homologous protein models, whose TM-score to the crystal structures is 0.46 (1a0iA) and 0.57 (2aotA). For these structures of 1a0iA and 2aotA, the distance between experimental and predicted pocket center (MCC calculated over binding residues) is 2.92 Å (0.60) and 1.97 Å (0.61), respectively. Because of structural distortions in the target models, both the matrix (Figures 3E) and the alignment (Figure 3F) slightly differ from those generated using crystal structures; however, *e*MatchSite still aligns binding residues with a Cα-RMSD of 2.70 Å. According to this alignment, both binding ligands adopt a similar orientation, which is shown in Figure 3H. These case studies illustrate the procedure implemented in *e*MatchSite and demonstrate that biologically correct sequence order-independent alignments of ligand binding sites can be constructed using protein models.

### Performance on the SOIPPA dataset

**Figure 4:**
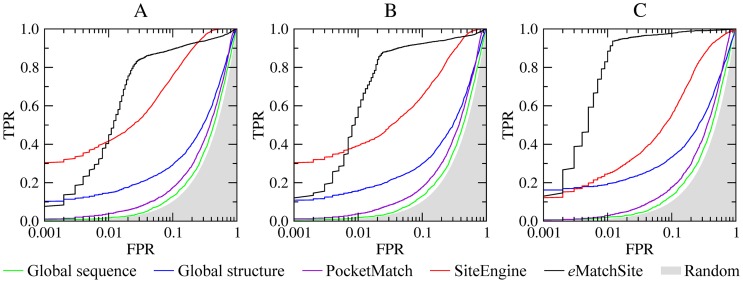
Performance of *e*MatchSite, PocketMatch and SiteEngine on the SOIPPA dataset of adenine-binding proteins. The accuracy of local alignment predictors is compared to that using global sequence and structure alignments for (**A**) crystal target structures, (**B**) high-, and (**C**) moderate-quality protein models. TPR and FPR are the true and false positive rates, respectively; gray area corresponds to a random prediction.

The first comparative assessment of the performance of *e*MatchSite in recognizing similar binding sites in globally dissimilar proteins is evaluated on the SOIPPA dataset of adenine-binding proteins [25]. In addition to target crystal structures, we perform binding site matching calculations using high- and moderate-quality protein models as well as distorted conformations. Receiver operating characteristics (ROC) are plotted in Figures 4 and S4 to evaluate the performance of binding site alignment algorithms, *e*MatchSite, SiteEngine and PocketMatch, in comparison to global similarity-based approaches (the corresponding AUC values are reported in Table S3). Using global sequence similarity yields an area under the ROC curve (AUC) of 0.55–0.56 across all target structures. As expected, these results are close to random, since the target proteins were selected based on the low pairwise global sequence similarity [25]. Structure alignments produce slightly better results with the AUC of 0.657, 0.655 and 0.671 for crystal structures, high- and low-quality models, respectively, indicating that adenine-binding proteins are slightly more similar at the global structure level compared to control proteins. In contrast, the AUC for *e*MatchSite, SiteEngine and PocketMatch using crystal structures is 0.941, 0.933 and 0.603, respectively; thus *e*MatchSite and SiteEngine perform comparably well, more efficiently detecting similar binding sites than PocketMatch. When high- (moderate-) quality protein models are used, the AUC for *e*MatchSite, SiteEngine and PocketMatch is 0.953 (0.987), 0.893 (0.856) and 0.615 (0.627), respectively. We note that the SOIPPA datasets of crystal structures and protein models comprise different numbers of proteins. This is because for some non-native target conformations, ligand binding sites were not predicted with an acceptable accuracy by *e*FindSite due to the deformations of their global structures. Nevertheless, binding pocket matching algorithms can still be compared to each other across the same set of target structures. On that account, the AUC for *e*MatchSite is 6% (13.1%) higher than that for SiteEngine using high- (moderate-) quality protein models.

Next, we assess the accuracy of the actual alignments of ligand binding sites between adenine-binding proteins. The performance comparison for *e*MatchSite and SOIPPA in matching adenine-binding sites is shown in Table 3. Here, the accuracy is evaluated by an RMSD calculated over ligand heavy atoms upon the superposition of aligned binding residues; correct alignments are defined as those upon which binding ligands are positioned within 2 Å and 5 Å RMSD. Using crystal structures, *e*MatchSite generates almost three times more accurate alignments than SOIPPA. Furthermore, *e*MatchSite maintains its capabilities to construct highly accurate alignments even when protein models of varying quality are used. Table 3 shows that depending on the model quality, the percentage of correctly aligned pairs of adenine-binding sites is only 4–9% lower than those aligned using crystal structures. This is an impressive result, given that the average Cα-RMSD from native calculated over ligand binding residues is 2.0–5.7 Å (Table 1). In addition to SOIPPA, we also compare the performance of *e*MatchSite to SiteEngine across different conformations of adenine-binding proteins. Table 4 reports the average ligand heavy-atom RMSD calculated upon the superposition of aligned binding residues (Table S5 shows the alignment accuracy separately for different ligands). *e*MatchSite systematically generates more accurate local alignments than SiteEngine, with the ligand RMSD better by roughly 1.0 Å, 1.5 Å and 2.5 Å when crystal structures, high- and moderate-quality models are used, respectively. These results demonstrate that *e*MatchSite not only constructs more accurate sequence order-independent binding site alignments, but also offers an improved tolerance to structural deformations in non-native protein structures.

**Table 3:** Comparison of sequence order-independent binding site alignments constructed by SOIPPA and *e*MatchSite for adenine-binding proteins.

Algorithm	RMSD threshold	Crystal structures	High-quality models	Moderate-quality models
SOIPPA	2 Å	6.3%	-	-
*e*MatchSite		15.6%	11.5%	6.5%
SOIPPA	5 Å	23.6%	-	-
*e*MatchSite		60.7%	56.4%	52.4%

The alignment accuracy is assessed by a ligand heavy-atom RMSD calculated upon the superposition of aligned binding residues. The percentage of benchmarking protein pairs for which the ligand RMSD is below 2 Å and 5 Å is reported.

**Table 4:** Comparison of sequence order-independent binding site alignments constructed by SiteEngine and *e*MatchSite for adenine-binding proteins from the SOIPPA dataset.

Algorithm	Crystal structures		High-quality models		Moderate-quality models	
	*RMSD*	Δ*RMSD* a	*RMSD*	Δ*RMSD* a	*RMSD*	Δ*RMSD* a
SiteEngine	5.63±3.37	3.67±2.91	6.78±3.29	4.83±2.92	7.89±3.68	6.01±3.46
*e*MatchSite	4.81±2.62	2.85±2.40	5.21±2.55	3.26±2.33	5.32±2.48	3.44±2.22

The alignment accuracy is assessed by the average ±standard deviation ligand heavy-atom RMSD calculated upon the superposition of aligned binding residues.

a Δ*RMSD* is calculated by subtracting from RMSD a ligand heavy-atom root-mean-square deviation upon the superposition of two ligands.

### Performance on the Kahraman and Homogeneous datasets

**Figure 5:**
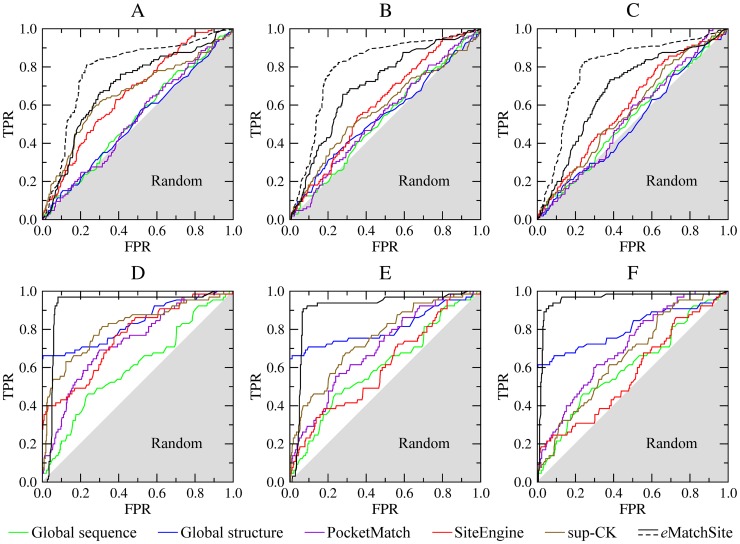
Performance comparison for *e*MatchSite, PocketMatch, SiteEngine and sup-CK. Binding site matching is conducted using the (**A**–**C**) Kahraman and (**D**–**F**) Homogeneous datasets. The accuracy of local alignment predictors is compared to that using global sequence and structure alignments for (**A**, **D**) crystal target structures, (**B**, **E**) high-, and (**C**, **F**) moderate-quality protein models. TPR and FPR are the true and false positive rates, respectively; gray area corresponds to a random prediction.

In the next assessment, we use the Kahraman and Homogeneous datasets compiled previously to evaluate the performance of binding site matching algorithms. The Kahraman dataset comprises proteins complexed with ligands of different sizes and physicochemical properties [42], whereas the Homogeneous dataset consists of ligands whose molecular weights are comparable [35]. Similar to the SOIPPA dataset, we use three conformations of the target proteins, crystal structures as well as high- and moderate-quality models (their characteristics are summarized in Table S1). Figure 5 shows the performance assessment for *e*MatchSite compared to two global similarity-based approaches as well as three binding site matching algorithms, PocketMatch, SiteEngine and sup-CK (the corresponding AUC values are reported in Table S3). Using the Kahraman dataset, the performance of PocketMatch is comparable to the global sequence and structure alignments and only marginally better than random. The accuracy of sup-CK is similar to SiteEngine, however, the latter performs slightly better using modeled target structures. What stands out is that *e*MatchSite systematically outperforms both sup-CK and SiteEngine with the AUC larger by 3–4% for the crystal structures and by 8–12% for protein models. In the original Kahraman dataset, three ligands that contain the adenine moiety, ATP, ADP and NAD, are considered as different molecules, thus recognizing a significant similarity between, for example ATP and ADP binding sites, counts as false positives. Therefore, similar to the SOIPPA dataset, we also assess the performance of *e*MatchSite for adenine-binding pockets grouped together, which is shown as dashed black lines in Figures 5A–C. Using this classification, the corresponding AUC for crystal structures, high- and moderate-quality models increases to 0.786, 0.799 and 0.792, respectively. This represents roughly a 10% improvement with respect to the original classification, suggesting that *e*MatchSite correctly recognizes similarities between different ligands containing the adenine moiety. Note that similar relationships were detected by applying MED-SuMo to purine-binding proteins from the PDB [29]. The classification of their binding sites revealed a number of distinct clusters, many of which are heterogeneous, i.e. linked to various kinds of purine-containing ligands. Finally, we analyze separately adenine-binding and other proteins from the Kahraman dataset. Figure S5 shows that *e*MatchSite gives the best performance for both subsets across different quality target structures.

The global structures of proteins binding different ligands in the Homogeneous dataset are notably more similar to each other than those from the Kahraman dataset. This explains a fairly high accuracy of global structure alignments shown in Figures 5D–F for the target crystal structures, high- and moderate-quality models; here, the corresponding AUC values are 0.835, 0.810 and 0.808, respectively (Table S3). The performance of PocketMatch, SiteEngine and sup-CK is similar, with the latter providing a slightly higher accuracy; however, it is still lower compared to the global structure alignments. In contrast, the accuracy of *e*MatchSite is significantly higher that using global as well as local alignment predictors. Furthermore, the performance differences increase when modeled structures are used as the targets; for instance, the AUC for *e*MatchSite is 11.2% (18.8%), 15.9% (21.0%) and 30.1% (25.0%) larger than that for sup-CK (PocketMatch) using crystal structures, high- and moderate-quality models, respectively.

### Performance on the Steroid dataset

**Figure 6:**
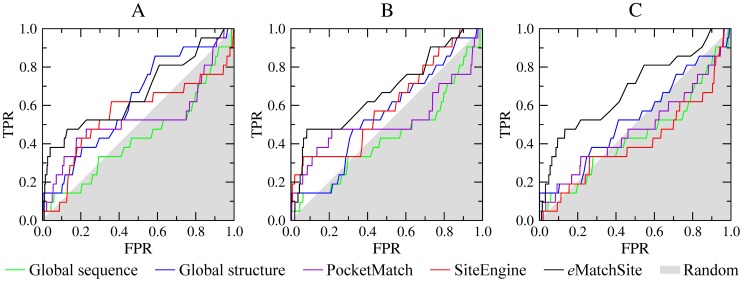
Performance of *e*MatchSite, PocketMatch and SiteEngine on the Steroid dataset. The accuracy of local alignment predictors is compared to that using global sequence and structure alignments for (**A**) crystal target structures, (**B**) high-, and (**C**) moderate-quality protein models. TPR and FPR are the true and false positive rates, respectively; gray area corresponds to a random prediction.

The last comparison is carried out using a dataset of steroid-binding proteins and a large set of control proteins that bind chemically dissimilar ligands, whose size is comparable to that of estradiol. Figure 6 shows the performance of *e*MatchSite compared to two global similarity-based approaches as well as two binding site matching algorithms, PocketMatch and SiteEngine (the corresponding AUC values are reported in Table S3). As for the other datasets, we use three conformations of the target proteins, crystal structures, high- and moderate-quality models, which is shown in Figures 6A, 6B and 6C, respectively. Binding site matching approaches perform better than the sequence-based approach across all datasets of target structures. The accuracy of PocketMatch, SiteEngine and the structure-based approach are fairly comparable, except for the target crystal structures, for which the structure-based approach performs better than PocketMatch and SiteEngine. The AUC values for *e*MatchSite are notably higher than those for PocketMatch and SiteEngine by 7–15% using target crystal structures and high-quality models, and by 20–26% using moderate-quality models. These results are qualitatively similar to those obtained for the SOIPPA, Kahraman and Homogeneous datasets and further demonstrate that *e*MatchSite is less sensitive to structural distortions in target proteins compared to other approaches.

## Availability and Future Directions

In this study, we describe *e*MatchSite, a new method for calculating the sequence order-independent alignments of ligand binding sites in proteins. This approach employs a set of residue-level scores derived from evolutionarily related templates and machine learning to estimate inter-residue distances upon the optimal superposition of ligand-binding sites. From these distances, local binding site alignments are constructed by the Kuhn-Munkres algorithm. In addition to the alignments, *e*MatchSite provides a calibrated significance score, which effectively identifies those pockets binding chemically similar ligands regardless of any global sequence and structure similarities between the target proteins. Benchmarking calculations are performed using four datasets of globally unrelated proteins that bind similar ligands. Compared to several other algorithms for ligand binding site matching, *e*MatchSite offers two unique features. The first is a high tolerance to structural deformations in ligand binding regions in protein models. For example, *e*MatchSite generates accurate alignments of adenine-binding pockets in crystal structures for almost three times more benchmarking protein pairs than SOIPPA. Moreover, the percentage of correctly aligned pairs of adenine-binding sites in weakly homologous protein models is only 4–9% lower than those aligned using crystal structures. This represents a significant improvement over other algorithms, e.g. the performance of *e*MatchSite in recognizing similar binding sites is 6% and 13% higher than that for SiteEngine using high- and moderate-quality protein models, respectively. Many proteins are known to undergo conformational changes upon ligand binding, however, a high tolerance to structural distortions in protein models suggests that *e*MatchSite will work well with ligand-free experimental structures as well. The second feature is the applicability to predicted pockets that may contain inaccuracies in the definition of binding residues. In general, the accuracy of binding residue prediction depends on the quality of target structures [39], thus using better models results in more accurate local alignments of their binding sites. Moreover, using binding residues predicted by evolution/structure-based approaches, such as *e*FindSite [39], may yield better accuracy in pocket matching between members of highly conserved protein families. These residues correlate with the conserved aspects of molecular function and are independent on the size of a particular ligand that was co-crystallized with the target protein. In addition, if ligand binding occurs outside cavities in protein structures, the corresponding binding residues can still be correctly identified by *e*FindSite as long as these are functionally conserved across sets of evolutionarily related proteins. Since *e*MatchSite includes strong evolutionary components in its scoring function, we should expect more accurate results for those target proteins belonging to functionally conserved families with distinct ligand binding patterns.

Constructing biologically correct alignments using predicted ligand binding sites in protein models opens up the possibility of investigating drug-protein interaction networks for complete proteomes. The prospective systems-level applications of *e*MatchSite include the development of safer biopharmaceuticals with reduced side effects, polypharmacology and rational drug repositioning. *e*MatchSite is freely available to the academic community as a web-server and a stand-alone software package at http://www.brylinski.org/ematchsite. This website also provides a complete documentation including walkthrough tutorials and case studies demonstrating the installation and execution procedures as well as the interpretation of results.

## Supporting Information

Figure S1 Effects of target structure distortions on the quality of local ligand binding site alignments. MCC is Matthew's correlation coefficient calculated against the reference alignments constructed using target crystal structures. Alignment accuracy is assessed separately for different ligands from the SOIPPA dataset: (**A**) ADP, (**B**) ATP, (**C**) FAD, (**D**) NAD, (**E**) SAH, and (**F**) SAM.
(TIF)

Figure S2 Correlation between the actual pairwise Cα-Cα distances upon the reference alignment of binding sites and those predicted by SVR. The correlation is plotted separately for different ligands from the SOIPPA dataset, ADP, ATP, FAD, NAD, SAH, and SAM, using (**A**) target crystal structures, (**B**) high- and (**C**) moderate-quality models, as well as structures distorted to (**D**) 2 Å, (**E**) 4 Å and (**F**) 6 Å Cα-RMSD.
(TIF)

Figure S3 ROC plots for the prediction of equivalent residue pairs using SVC and different quality target structures. The accuracy is assessed separately for different ligands from the SOIPPA dataset, (**A**) ADP, (**B**) ATP, (**C**) FAD, (**D**) NAD, (**E**) SAH, and (**F**) SAM. TPR and FPR are the true and false positive rates, respectively; gray area corresponds to a random prediction.
(TIF)

Figure S4 Performance of *e*MatchSite, PocketMatch and SiteEngine on the SOIPPA dataset of adenine-binding proteins. The accuracy of local alignment predictors is compared to that using global sequence and structure alignments for (**A**) crystal target structures, (**B**) high- and (**C**) moderate-quality protein models, as well as structures distorted to (**D**) 2 Å, (**E**) 4 Å and (**F**) 6 Å Cα-RMSD. TPR and FPR are the true and false positive rates, respectively; gray area corresponds to a random prediction.
(TIF)

Figure S5 Performance of *e*MatchSite, PocketMatch, SiteEngine and sup-CK on the Kahraman dataset. Binding site matching is conducted using (**A**–**C**) adenine-binding and (**D**–**F**) other proteins. The accuracy of local alignment predictors is compared to that using global sequence and structure alignments for (**A**, **D**) crystal target structures, (**B**, **E**) high-, and (**C**, **F**) moderate-quality protein models. TPR and FPR are the true and false positive rates, respectively; gray area corresponds to a random prediction.
(TIF)

Table S1 Global and local structure quality of benchmarking proteins from the SOIPPA, Kahraman, Homogeneous and Steroid datasets. High- and moderate-quality models are constructed by *e*Thread. Distorted structures were generated by deforming the crystal structures to a desired Cα-RMSD. Ligand binding sites and residues are detected by *e*FindSite.
(PDF)

Table S2 Accuracy of inter-residue distance prediction for adenine-binding proteins from the SOIPPA dataset. The Pearson correlation coefficient (PCC) and the mean squared error (MSE) are calculated for the actual pairwise Cα-Cα distances upon the superposition of binding ligands and those predicted by SVR from residue-level scores. The accuracy is reported separately for different binding ligands and target protein conformations including crystal structures, high- and moderate-quality protein models, as well as structures distorted to 2 Å, 4 Å and 6 Å Cα-RMSD.
(PDF)

Table S3 Performance of *e*MatchSite, PocketMatch, SiteEngine and sup-CK in recognizing similar ligand binding sites. The accuracy is assessed by the area under ROC. The performance of local alignment predictors is compared to that using global sequence and structure alignments for different target structures from the SOIPPA, Kahraman, Homogeneous and Steroid datasets.
(PDF)

Table S4 Comparison of sequence order-independent binding site alignments constructed by SOIPPA and *e*MatchSite for adenine-binding proteins. The alignment accuracy is assessed by a ligand heavy-atom RMSD calculated upon the superposition of aligned binding residues. The percentage of benchmarking protein pairs for which the RMSD is below 2 Å and 5 Å is reported.
(PDF)

Table S5 Comparison of sequence order-independent binding site alignments constructed by SiteEngine and *e*MatchSite for adenine-binding proteins from the SOIPPA dataset. The alignment accuracy is assessed by the average ±standard deviation ligand heavy-atom RMSD calculated upon the superposition of aligned binding residues.
(PDF)
