# Supplementary figures and images for: eMatchSite: Sequence Order-Independent Structure Alignments of Ligand Binding Pockets in Protein Models
Source: PLoS Comput Biol. 2014 Sep 18;10(9):e1003829. doi: 10.1371/journal.pcbi.1003829 (PMC4168975; doi:10.1371/journal.pcbi.1003829)

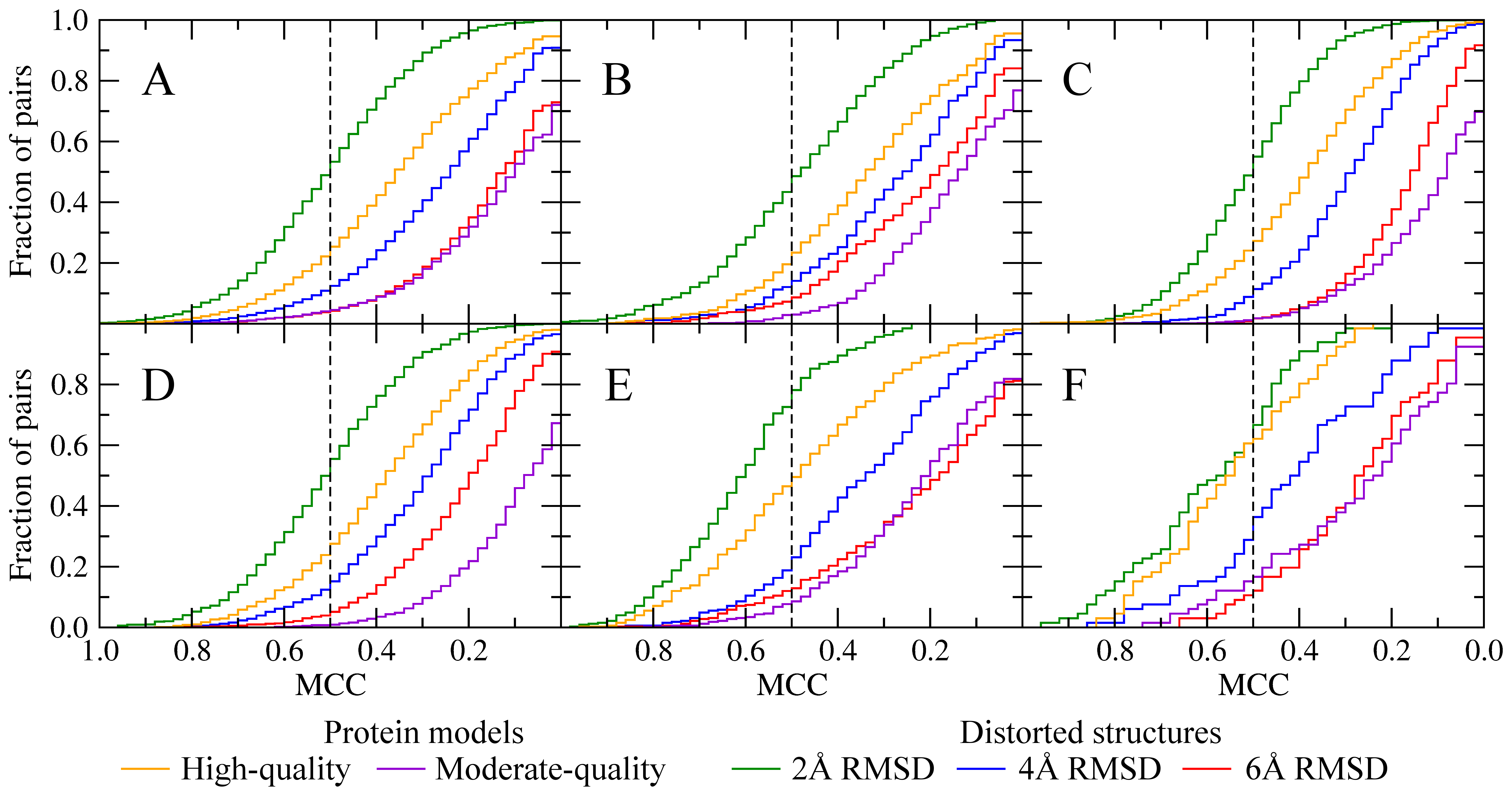

Supplement: Figure S1 — Effects of target structure distortions on the quality of local ligand binding site alignments. MCC is Matthew's correlation coefficient calculated against the reference alignments constructed using target crystal structures. Alignment accuracy is assessed separately for different ligands from the SOIPPA dataset: (A) ADP, (B) ATP, (C) FAD, (D) NAD, (E) SAH, and (F) SAM. (TIF) [file pcbi.1003829.s001.tif]

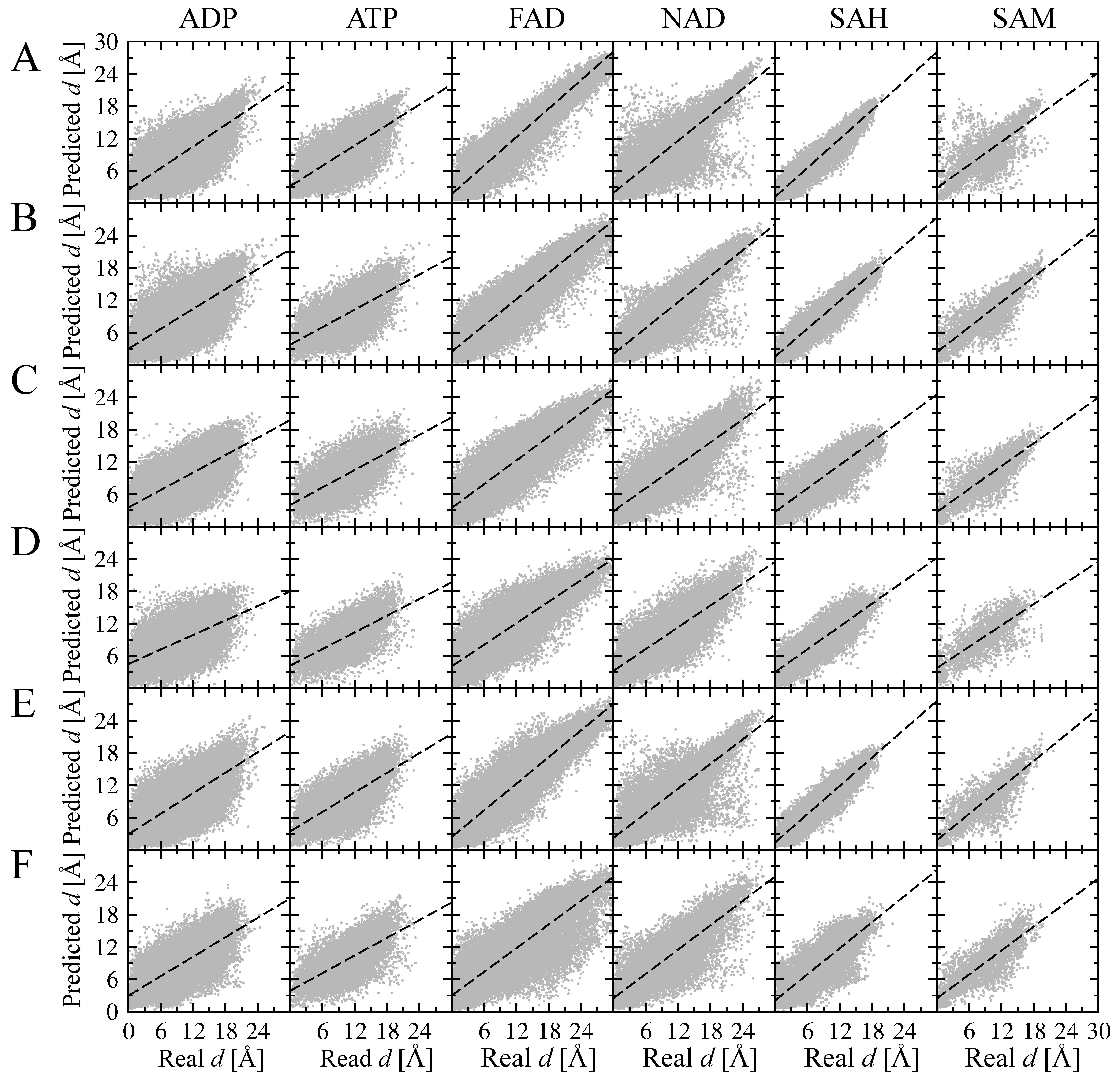

Supplement: Figure S2 — Correlation between the actual pairwise Cα-Cα distances upon the reference alignment of binding sites and those predicted by SVR. The correlation is plotted separately for different ligands from the SOIPPA dataset, ADP, ATP, FAD, NAD, SAH, and SAM, using (A) target crystal structures, (B) high- and (C) moderate-quality models, as well as structures distorted to (D) 2 Å, (E) 4 Å and (F) 6 Å Cα-RMSD. (TIF) [file pcbi.1003829.s002.tif]

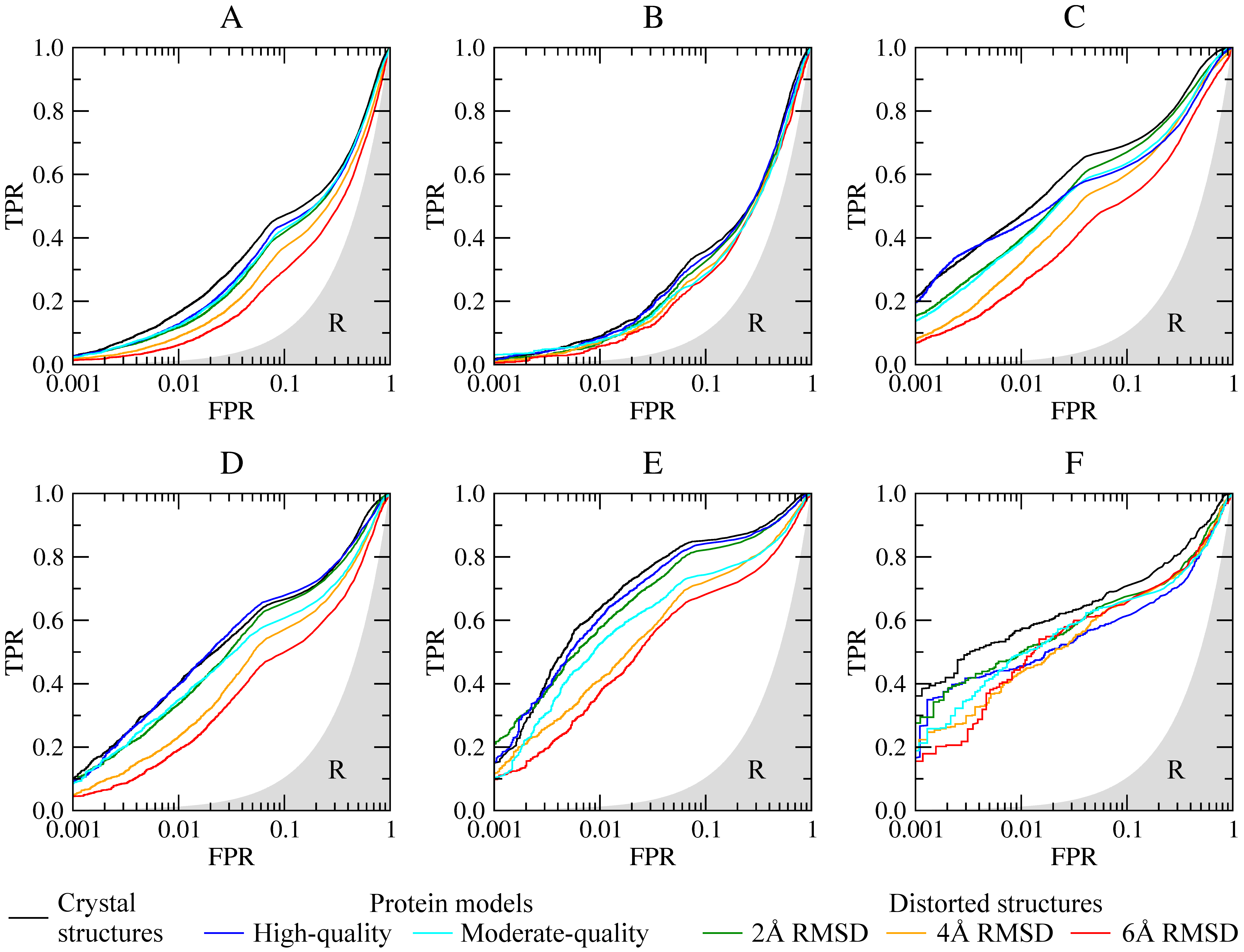

Supplement: Figure S3 — ROC plots for the prediction of equivalent residue pairs using SVC and different quality target structures. The accuracy is assessed separately for different ligands from the SOIPPA dataset, (A) ADP, (B) ATP, (C) FAD, (D) NAD, (E) SAH, and (F) SAM. TPR and FPR are the true and false positive rates, respectively; gray area corresponds to a random prediction. (TIF) [file pcbi.1003829.s003.tif]

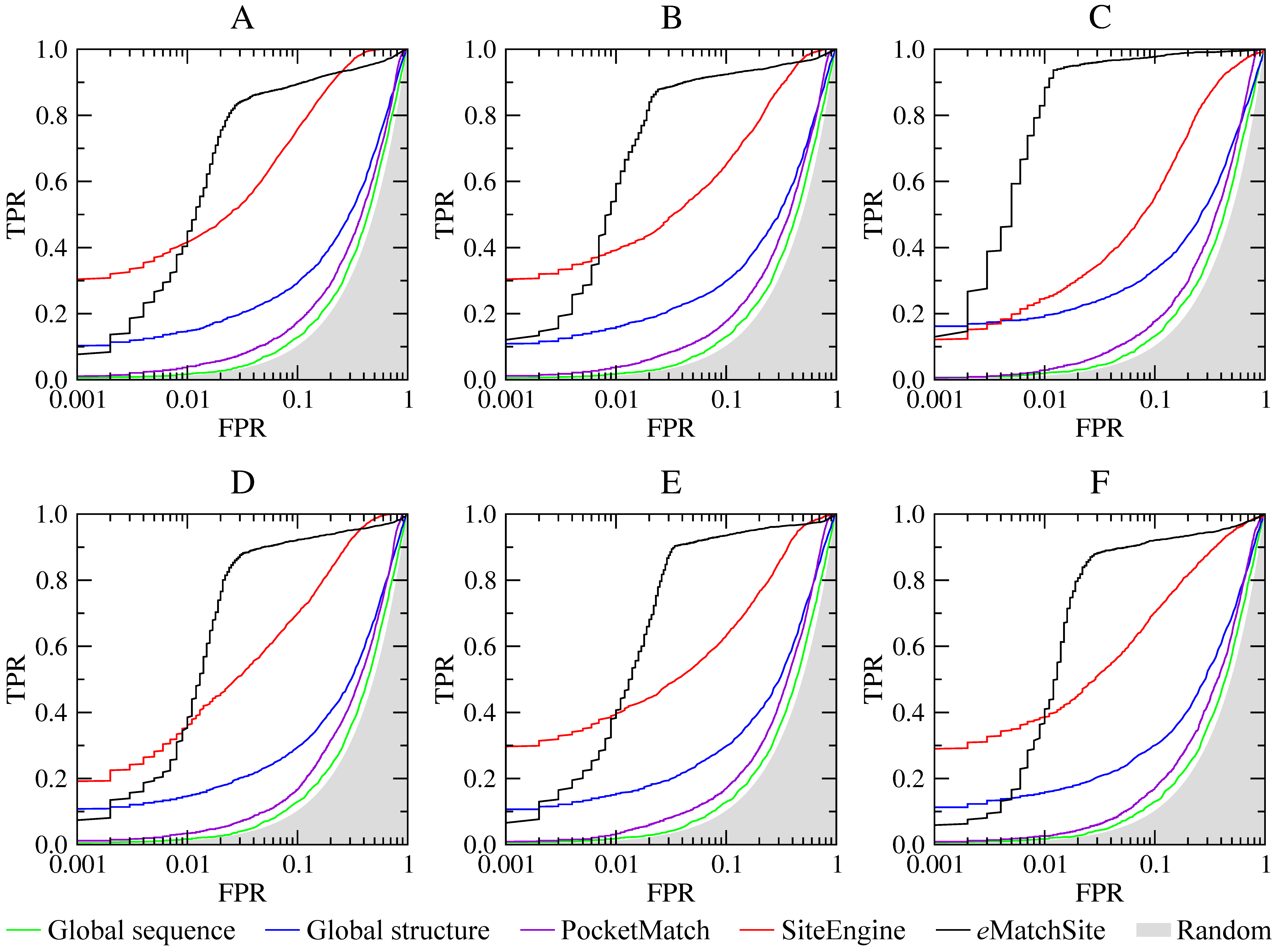

Supplement: Figure S4 — Performance of eMatchSite, PocketMatch and SiteEngine on the SOIPPA dataset of adenine-binding proteins. The accuracy of local alignment predictors is compared to that using global sequence and structure alignments for (A) crystal target structures, (B) high- and (C) moderate-quality protein models, as well as structures distorted to (D) 2 Å, (E) 4 Å and (F) 6 Å Cα-RMSD. TPR and FPR are the true and false positive rates, respectively; gray area corresponds to a random prediction. (TIF) [file pcbi.1003829.s004.tif]

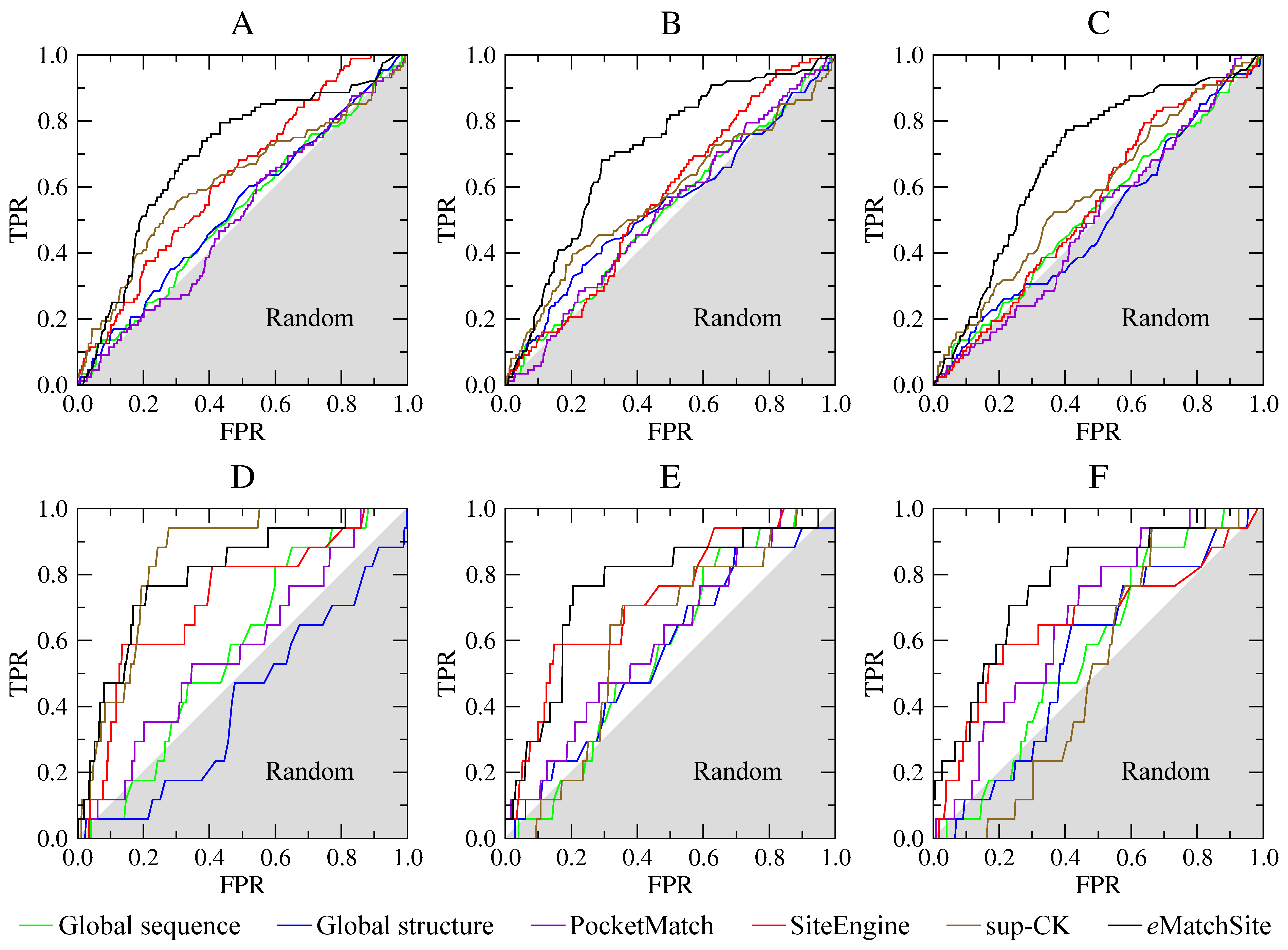

Supplement: Figure S5 — Performance of eMatchSite, PocketMatch, SiteEngine and sup-CK on the Kahraman dataset. Binding site matching is conducted using (A–C) adenine-binding and (D–F) other proteins. The accuracy of local alignment predictors is compared to that using global sequence and structure alignments for (A, D) crystal target structures, (B, E) high-, and (C, F) moderate-quality protein models. TPR and FPR are the true and false positive rates, respectively; gray area corresponds to a random prediction. (TIF) [file pcbi.1003829.s005.tif]
