# Supplementary material for: eMatchSite: Sequence Order-Independent Structure Alignments of Ligand Binding Pockets in Protein Models
Source: PLoS Comput Biol. 2014 Sep 18;10(9):e1003829. doi: 10.1371/journal.pcbi.1003829 (PMC4168975; doi:10.1371/journal.pcbi.1003829)
Supplement: Table S1 — Global and local structure quality of benchmarking proteins from the SOIPPA, Kahraman, Homogeneous and Steroid datasets. High- and moderate-quality models are constructed by eThread. Distorted structures were generated by deforming the crystal structures to a desired Cα-RMSD. Ligand binding sites and residues are detected by eFindSite. (PDF) [file pcbi.1003829.s006.pdf]

Dataset	Number of targets	Global structure				Ligand binding pocket		
		Cα-RMSD [Å]	*TM-score*	*MaxSub*	*GDT-score*	*RMSD[e] [Å]*	*Distance[f] [Å]*	*MCC[g]*
SOIPPA								
Crystal[a]	211	-	-	-	-	-	1.7 ±1.4	0.70 ±0.10
HQ models[b]	202	4.4 ±2.4	0.83 ±0.07	0.65 ±0.12	0.69 ±0.10	2.0 ±1.4	1.8 ±1.4	0.67 ±0.10
MQ models[c]	174	13.2 ±4.6	0.54 ±0.10	0.34 ±0.10	0.39 ±0.09	5.7 ±3.5	1.9 ±1.3	0.62 ±0.10
2Å RMSD[d]	205	2.0 ±0.1	0.91 ±0.03	0.78 ±0.02	0.78 ±0.03	1.3 ±0.5	1.8 ±1.2	0.69 ±0.10
4Å RMSD[d]	199	4.0 ±0.1	0.78 ±0.05	0.55 ±0.06	0.63 ±0.04	2.4 ±1.0	2.1 ±1.3	0.64 ±0.10
6Å RMSD[d]	174	6.0 ±0.1	0.68 ±0.06	0.46 ±0.07	0.54 ±0.05	3.2 ±1.5	2.5 ±1.5	0.62 ±0.10
Kahraman								
Crystal[a]	53	-	-	-	-	-	2.0 ±1.3	0.65 ±0.11
HQ models[b]	53	4.5 ±2.6	0.84 ±0.08	0.66 ±0.14	0.70 ±0.12	1.8 ±1.3	2.2 ±1.5	0.63 ±0.11
MQ models[c]	40	13.5 ±6.2	0.59 ±0.10	0.39 ±0.11	0.43 ±0.10	5.4 ±3.5	2.2 ±1.4	0.59 ±0.10
Homogeneous								
Crystal[a]	51	-	-	-	-	-	3.2 ±1.8	0.63 ±0.14
HQ models[b]	47	5.1 ±3.3	0.82 ±0.08	0.65 ±0.12	0.68 ±0.10	2.2 ±2.0	3.2 ±2.0	0.62 ±0.13
MQ models[c]	29	12.3 ±4.3	0.56 ±0.10	0.36 ±0.10	0.42 ±0.10	4.3 ±3.6	2.9 ±1.8	0.59 ±0.11
Steroid								
Crystal[a]	1780	-	-	-	-	-	2.3 ±1.5	0.67 ±0.13
HQ models[b]	1660	4.4 ±2.4	0.84 ±0.06	0.69 ±0.11	0.71 ±0.09	2.0 ±1.8	2.3 ±1.5	0.65 ±0.13
MQ models[c]	1153	12.7 ±4.4	0.54 ±0.10	0.35 ±0.10	0.40 ±0.10	5.5 ±3.5	2.5 ±1.5	0.61 ±0.12

[a] Crystal structures. [b] High-quality protein models. [c] Moderate-quality protein models. [d] Structures artificially distorted to a desired Cα-RMSD. [e] Heavy-atom RMSD calculated over binding residues. [f] Distance between predicted pocket center and the geometric center of bound ligand. [g] Matthew's correlation coefficient for predicted binding residues.
