# Supplementary material for: eMatchSite: Sequence Order-Independent Structure Alignments of Ligand Binding Pockets in Protein Models
Source: PLoS Comput Biol. 2014 Sep 18;10(9):e1003829. doi: 10.1371/journal.pcbi.1003829 (PMC4168975; doi:10.1371/journal.pcbi.1003829)
Supplement: Table S2 — Accuracy of inter-residue distance prediction for adenine-binding proteins from the SOIPPA dataset. The Pearson correlation coefficient (PCC) and the mean squared error (MSE) are calculated for the actual pairwise Cα-Cα distances upon the superposition of binding ligands and those predicted by SVR from residue-level scores. The accuracy is reported separately for different binding ligands and target protein conformations including crystal structures, high- and moderate-quality protein models, as well as structures distorted to 2 Å, 4 Å and 6 Å Cα-RMSD. (PDF) [file pcbi.1003829.s007.pdf]

Structure dataset	Binding ligand											
	ADP		***ATP***		***FAD***		***NAD***		***SAH***		***SAM***	
	PCC[a]	*MSE*[b]	*PCC*[a]	*MSE*[b]	*PCC*[a]	*MSE*[b]	*PCC*[a]	*MSE*[b]	*PCC*[a]	*MSE*[b]	*PCC*[a]	*MSE*[b]
Crystal structures	0.79	6.4	0.73	8.1	0.96	2.4	0.89	4.6	0.95	1.6	0.76	7.2
High-quality models	0.75	7.3	0.72	8.6	0.92	4.6	0.86	6.1	0.94	1.9	0.88	3.6
Moderate-quality models	0.75	7.9	0.68	10.1	0.88	7.2	0.86	6.3	0.86	4.1	0.83	4.9
Distorted to 2Å RMSD	0.75	7.6	0.68	9.6	0.93	4.3	0.88	5.2	0.94	2.0	0.86	4.4
Distorted to 4Å RMSD	0.69	8.7	0.68	9.7	0.88	6.7	0.84	6.9	0.86	4.4	0.83	4.9
Distorted to 6Å RMSD	0.60	10.9	0.68	10.0	0.83	8.5	0.81	7.3	0.85	4.4	0.76	7.5

[a] Pearson correlation coefficient. [b] Mean squared error in Å.
