# Supplementary material for: eMatchSite: Sequence Order-Independent Structure Alignments of Ligand Binding Pockets in Protein Models
Source: PLoS Comput Biol. 2014 Sep 18;10(9):e1003829. doi: 10.1371/journal.pcbi.1003829 (PMC4168975; doi:10.1371/journal.pcbi.1003829)
Supplement: Table S3 — Performance of eMatchSite, PocketMatch, SiteEngine and sup-CK in recognizing similar ligand binding sites. The accuracy is assessed by the area under ROC. The performance of local alignment predictors is compared to that using global sequence and structure alignments for different target structures from the SOIPPA, Kahraman, Homogeneous and Steroid datasets. (PDF) [file pcbi.1003829.s008.pdf]

Dataset	Global similarity		Binding site matching			
	Sequence	***Structure***	***eMatchSite***	***PocketMatch***	***SiteEngine***	***sup-CK***
SOIPPA						
Crystal[a]	0.553	0.657	0.941	0.603	0.933	-
HQ models[b]	0.553	0.655	0.953	0.615	0.893	-
MQ models[c]	0.564	0.671	0.987	0.627	0.856	-
2Å RMSD[d]	0.553	0.653	0.948	0.616	0.911	-
4Å RMSD[d]	0.553	0.659	0.954	0.622	0.880	-
6Å RMSD[d]	0.556	0.660	0.948	0.610	0.889	-
Kahraman						
Crystal[a]	0.528	0.514	0.693	0.523	0.660	0.657
HQ models[b]	0.528	0.542	0.702	0.542	0.605	0.578
MQ models[c]	0.528	0.505	0.675	0.536	0.594	0.569
Homogeneous						
Crystal[a]	0.603	0.835	0.924	0.736	0.760	0.812
HQ models[b]	0.603	0.810	0.910	0.700	0.605	0.751
MQ models[c]	0.603	0.808	0.957	0.707	0.571	0.656
Steroid						
Crystal[a]	0.434	0.621	0.663	0.531	0.550	-
HQ models[b]	0.431	0.549	0.667	0.520	0.598	-
MQ models[c]	0.440	0.529	0.678	0.476	0.413	-

[a] Crystal structures. [b] High-quality protein models. [c] Moderate-quality protein models. [d] Structures artificially distorted to a desired Cα-RMSD.
