# Supplementary material for: eMatchSite: Sequence Order-Independent Structure Alignments of Ligand Binding Pockets in Protein Models
Source: PLoS Comput Biol. 2014 Sep 18;10(9):e1003829. doi: 10.1371/journal.pcbi.1003829 (PMC4168975; doi:10.1371/journal.pcbi.1003829)
Supplement: Table S4 — Comparison of sequence order-independent binding site alignments constructed by SOIPPA and eMatchSite for adenine-binding proteins. The alignment accuracy is assessed by a ligand heavy-atom RMSD calculated upon the superposition of aligned binding residues. The percentage of benchmarking protein pairs for which the RMSD is below 2 Å and 5 Å is reported. (PDF) [file pcbi.1003829.s009.pdf]

Algorithm	RMSD threshold	Crystal structures	Protein models		Distorted structures		
			High-quality	***Moderate-quality***	***2Å RMSD***	***4Å RMSD***	***6Å RMSD***
SOIPPA	2Å	6.3%	-	-	-	-	-
*e*MatchSite		15.6%	11.5%	6.5%	12.1%	7.3%	4.4%
SOIPPA	5Å	23.6%	-	-	-	-	-
*e*MatchSite		60.7%	56.4%	52.4%	59.5%	50.4%	40.0%
