# Supplementary material for: eMatchSite: Sequence Order-Independent Structure Alignments of Ligand Binding Pockets in Protein Models
Source: PLoS Comput Biol. 2014 Sep 18;10(9):e1003829. doi: 10.1371/journal.pcbi.1003829 (PMC4168975; doi:10.1371/journal.pcbi.1003829)
Supplement: Table S5 — Comparison of sequence order-independent binding site alignments constructed by SiteEngine and eMatchSite for adenine-binding proteins from the SOIPPA dataset. The alignment accuracy is assessed by the average ±standard deviation ligand heavy-atom RMSD calculated upon the superposition of aligned binding residues. (PDF) [file pcbi.1003829.s010.pdf]

Algorithm	Ligand	Crystal structures		**Protein models**				**Distorted structures**					
				High-quality		***Moderate-quality***		***2Å RMSD***		***4Å RMSD***		***6Å RMSD***	
		RMSD	Δ*RMSD*[a]	*RMSD*	Δ*RMSD*[a]	*RMSD*	Δ*RMSD*[a]	*RMSD*	Δ*RMSD*[a]	*RMSD*	Δ*RMSD*[a]	*RMSD*	Δ*RMSD*[a]
SiteEngine	ADP	5.5 ±2.3	3.9 ±2.2	6.3 ±2.2	4.7 ±2.1	6.6 ±2.2	5.1 ±2.2	6.2 ±2.2	4.6 ±2.2	6.8 ±2.1	5.2 ±2.0	8.4 ±12.8	6.9 ±12.8
*e*MatchSite		5.1 ±2.4	3.4 ±2.3	5.4 ±2.3	3.8 ±2.3	5.3 ±2.4	3.8 ±2.4	5.1 ±2.2	3.5 ±2.1	6.0 ±2.3	4.4 ±2.2	6.6 ±2.7	5.0 ±2.7
SiteEngine	ATP	6.2 ±2.3	3.9 ±2.2	6.9 ±1.9	4.5 ±1.9	7.2 ±2.1	4.8 ±2.1	7.1 ±2.3	4.8 ±2.3	7.0 ±2.2	4.7 ±2.1	7.5 ±2.3	5.1 ±2.3
*e*MatchSite		6.0 ±2.3	3.7 ±2.2	6.0 ±2.2	3.6 ±2.0	6.2 ±1.9	3.8 ±1.9	5.6 ±2.0	3.3 ±1.9	6.7 ±2.6	4.3 ±2.6	5.8 ±1.8	3.4 ±1.8
SiteEngine	FAD	6.8 ±4.8	4.1 ±4.0	8.3 ±4.6	5.6 ±4.0	10.5 ±4.5	8.0 ±4.5	7.3 ±4.4	4.7 ±3.8	8.4 ±4.5	5.7 ±3.9	9.5 ±4.2	6.7 ±3.9
*e*MatchSite		4.5 ±2.4	1.8 ±1.7	5.1 ±2.2	2.4 ±1.7	5.6 ±2.4	3.2 ±2.0	4.8 ±2.3	2.1 ±1.7	5.8 ±2.6	3.1 ±2.0	7.4 ±3.3	4.7 ±2.8
SiteEngine	NAD	5.7 ±3.9	3.3 ±3.4	7.4 ±3.9	5.1 ±3.6	8.4 ±3.7	6.0 ±3.6	6.5 ±3.8	4.1 ±3.4	7.6 ±3.5	5.3 ±3.4	8.8 ±3.6	6.3 ±3.3
*e*MatchSite		5.1 ±3.4	2.7 ±3.1	5.7 ±3.6	3.3 ±3.3	5.9 ±2.9	3.6 ±2.6	4.8 ±3.2	2.5 ±2.8	5.4 ±2.7	3.1 ±2.5	2.9 ±2.4	3.4 ±2.1
SiteEngine	SAH	2.3 ±1.7	1.3 ±1.2	4.6 ±2.9	3.6 ±2.8	6.3 ±3.3	5.5 ±3.3	3.5 ±2.0	2.5 ±1.8	5.5 ±2.8	4.5 ±2.7	6.4 ±2.7	5.4 ±2.8
*e*MatchSite		2.2 ±1.1	1.2 ±0.7	2.7 ±1.4	1.7 ±1.2	3.3 ±1.2	2.4 ±1.2	2.6 ±0.9	1.6 ±0.7	3.3 ±1.5	2.3 ±1.4	4.7 ±2.1	3.7 ±2.1
SiteEngine	SAM	3.9 ±3.0	2.3 ±2.3	5.3 ±3.2	3.8 ±3.0	7.4 ±2.7	6.0 ±2.6	3.8 ±2.7	2.4 ±2.0	5.1 ±2.4	3.7 ±2.0	5.7 ±2.8	4.4 ±2.5
*e*MatchSite		5.0 ±3.4	3.4 ±3.1	3.0 ±2.1	1.6 ±1.1	4.1 ±2.0	2.7 ±1.5	3.2 ±2.1	1.8 ±1.3	3.8 ±1.8	2.4 ±1.3	4.2 ±2.6	2.9 ±2.0
SiteEngine	All	5.6 ±3.4	3.7 ±2.9	6.8 ±3.3	4.8 ±2.9	7.9 ±3.7	6.0 ±3.5	6.4 ±3.2	4.4 ±2.8	7.2 ±3.1	5.2 ±2.8	8.5 ±8.6	6.5 ±8.5
*e*MatchSite		4.8 ±2.6	2.8 ±2.4	5.2 ±2.6	3.3 ±2.3	5.3 ±2.5	3.4 ±2.2	4.8 ±2.4	2.9 ±2.2	5.7 ±2.5	3.8 ±2.3	6.5 ±2.9	4.4 ±2.6

[a] Δ*RMSD* is calculated by subtracting from RMSD a ligand heavy-atom root-mean-square deviation upon the superposition of two ligands.
